# (Re)defining urban villages and their potential in sustaining local authenticity: A case study of Da Lat, Viet Nam

**DOI:** 10.1371/journal.pone.0345741

**Published:** 2026-04-03

**Authors:** Khoa Anh Doan

**Affiliations:** Urban Planning Department, University of Architecture Ho Chi Minh city, Xuan Hoa Ward, Ho Chi Minh City, Viet Nam; Hunan University, CHINA

## Abstract

Urban villages have been theorised in three dominant ways: as ethnic enclaves in migration studies, as the UK Urban Village Concept, and as villages-in-the-city in the Global South. Whether these interpretations can coexist within a single urban context remains insufficiently examined. This study addresses this gap through Da Lat, Viet Nam, where rapid urbanisation and the state-led Green Urban Village pilot project have brought these models into direct interaction. The study adopts a multidisciplinary, multi-method approach combining historical and literature review, spatial mapping, expert-based Delphi assessment, topic modelling, and multi-criteria evaluation using the Analytic Hierarchy Process with optimisation techniques. A total of 74 villages were assessed across eight evaluation criteria using a context-sensitive scoring framework. Findings confirm the coexistence of ViCs, core attributes associated with ethnic enclaves, and a future-oriented Urban Village Concept. Most existing villages scored positively, demonstrating strong socio-cultural and spatial embeddedness, while the ecological dimension remains underdeveloped. The Green Urban Village pilot aligns with environmental objectives but shows weak contextual integration. A scenario-based integration with an adjacent village yields a more balanced performance across all criteria. The study empirically demonstrates the coexistence of hybrid urban village forms and analytically illustrates how a relational approach – here referred to as Green Urban Village 2.0 – could anchor sustainability-oriented planning within existing village structures. The findings contribute to urban village theory and offer context-sensitive insights for sustainable neighbourhood planning in Viet Nam and comparable settings.

## Introduction

Since its introduction by American sociologist Herbert Gans in 1960, the concept of the *urban village* has been widely mobilised across sociology, planning, and urban studies to explain community cohesion, neighbourhood sustainability, and informal urbanisation. Despite its popularity, the term remains conceptually ambiguous and highly context-dependent, encompassing diverse – and sometimes contradictory – meanings across different geographical and disciplinary traditions.

Existing literature generally identifies three dominant interpretations. First, within migration and urban sociology, *urban villages* are understood as *ethnic enclaves* that sustain cultural identity and social networks within metropolitan environments. Second, in the United Kingdom (UK), the *Urban Village Concept* emerged as a normative planning model promoting compact, mixed-use, and socially cohesive neighbourhoods. Third, in the Global South, the *Villages-in-the-city* (ViCs) phenomenon describes rural-origin settlements embedded within rapidly urbanising areas. While the first and third interpretations are largely descriptive of socio-spatial processes, the UK model is explicitly prescriptive, embedding normative assumptions about community, place, and urban form.

The *Urban Village Concept* has also been subject to sustained critical scrutiny. Scholars have questioned its nostalgic idealisation of “traditional” community life, its tendency to universalise Eurocentric neighbourhood forms, and its potential exclusionary effects when translated into real estate-driven urban regeneration. Rather than fostering social diversity, such projects often privilege middle-class lifestyles and commodify local identity, raising concerns about the model’s transferability beyond its original context – particularly in cities shaped by informality and socio-cultural hybridity.

Against this backdrop, a critical question emerges: can multiple manifestations of the *urban village* – *ethnic enclaves*, *prescriptive planning models*, and *ViCs* – coexist within a single urban setting? Addressing this question is essential for understanding how global planning concepts interact with local histories, practices, and power relations in shaping sustainable urban futures.

This study examines Da Lat, a highland city in Lam Dong province, Viet Nam, where such dynamics have become increasingly visible, particularly following the introduction of the *Green Urban Village* pilot project in 2016. Originally developed as a French colonial hill station, Da Lat later experienced successive waves of Kinh migration, leading to the formation of village-based settlements within and around the city. Indigenous communities in the surrounding districts further contributed to a complex socio-spatial landscape. Although many settlements have been absorbed into the expanding urban fabric, several continue to preserve distinctive practices and spatial forms, including agricultural production, communal institutions, and ritual landscapes – resonating with interpretations of *urban villages* as both *ethnic enclaves* and *ViCs.*

The *Green Urban Village* model – apparently inspired by the *Urban Village Concept* – seeks to integrate high-tech agriculture, green development, and high-quality urban living. However, its prioritisation of vacant land and newly planned areas raises concerns regarding contextual sensitivity and engagement with existing village-based settlements. Together with the author’s positionality as a local researcher, these conditions motivate a critical re-examination of how *urban villages* are defined and operationalised in Da Lat.

Accordingly, this study hypothesises that multiple forms of *urban villages* coexist within Da Lat and its surrounding areas, potentially forming hybrid configurations that interact with the state-led *Green Urban Village* initiative. Focusing on the area defined by the *General Planning of Da Lat City and its vicinity to 2030, with a vision to 2050* (Decision No. 704/QD-TTg, 12 May 2014 – hereafter referred to as *Plan No. 704*), the research employs a multidisciplinary, multi-method approach to develop a framework for identifying *urban village* types, empirically demonstrate their coexistence, and assess their potential contributions to sustaining local authenticity and urban sustainability.

Specifically, the study addresses two research questions:

1. Do the three dominant interpretations of the *urban village* – *ethnic enclaves*, the *Urban Village Concept*, and ViCs – coexist within a single urban context such as Da Lat?
2. If so, how can these forms be integrated to inform the development of a more context-sensitive and sustainable model of the *Green Urban Village*?

By foregrounding hybridity and contextual specificity, the study contributes to debates on neighbourhood sustainability and challenges the uncritical transfer of normative planning models to cities in the Global South.

## Theoretical framework

### “Urban village” as an ethnic enclave

The concept of the *urban village* first emerged from sociological studies of *ethnic enclaves*, notably in Gans’ research on inner-city migrant communities. It refers to areas where successive generations of migrants from shared ethnic backgrounds concentrate, sustaining dense, localised social ties within metropolitan environments [1,2]. Empirical studies document such formations in diverse contexts, including Italian, German, Jewish, Lithuanian, and mixed-ethnic settlements in cities such as Sydney, Espírito Santo, Chicago, and Manhattan, as well as Little Saigon in southern California [1].

In these settings, *urban villages* function as socially cohesive environments shaped by kinship networks, cultural traditions, and collective practices. They provide supportive frameworks that facilitate migrants’ adaptation to urban life while maintaining symbolic and practical connections to places of origin. At the same time, these enclaves may remain partially socially segregated from the wider urban context [2].

Although many historic *ethnic enclaves* have been transformed or displaced by inner-city redevelopment, new ones continue to emerge through ongoing migration. Their persistence highlights forms of urban authenticity grounded in everyday social practices and cultural continuity, reinforcing their relevance to debates on urban identity and inclusive regeneration.

### “Urban village” as a neighborhood planning concept

In the early 1980s, *Neo-traditionalism* emerged in the United States (US) as a response to Modernism and postwar urban planning. Approaches such as *New Urbanism*, *Traditional Neighbourhood Development*, *Transit-Oriented Development*, and *Smart Growth* promoted compact, walkable, mixed-use neighbourhoods as alternatives to dispersed urban form [3–6]. During the 1990s, growing environmental awareness and the adoption of *Agenda 21* further reinforced the emphasis on sustainable neighbourhood development, particularly in the UK and the US [7,8].

Within this context, the *Urban Village Concept* evolved through transatlantic exchanges between British and American planners, notably the *Urban Villages Group* and the *Congress for the New Urbanism* [9,10]. Aldous’ manifesto [11] framed the *urban village* as a compact, mixed-use neighbourhood capable of supporting everyday life within a human-scale environment. Drawing on urban design traditions associated with Jane Jacobs and Kevin Lynch [5,10,12], the model foregrounds density, functional mix, pedestrian-oriented public space, access to employment and services, and a strong sense of place [6].

Distinctively, the concept aspires to restore a degree of social and functional self-sufficiency associated with traditional villages. Although proponents recognise that the term “village” is partly metaphorical, it deliberately invokes ideals of stability and community cohesion as planning objectives [10,11].

Nevertheless, the concept has been widely criticised for its nostalgic framing, normative assumptions about community, and limited sensitivity to social inequality and local socio-cultural diversity. Critics argue that urban-village rhetoric often mobilises nostalgia to legitimise regeneration strategies while obscuring displacement and social exclusion [13]. Others highlight the gap between sustainability claims and the realities of commercially driven housing development, raising doubts about the model’s transferability across contexts [14]. From a cultural perspective, the “village” invoked in neotraditional planning has been interpreted as a form of spatial marketing that produces sanitised, nostalgic environments and may reinforce exclusionary identities [15]. Together, these critiques caution against the uncritical application of the *Urban Village Concept* beyond its original socio-spatial settings.

### “Urban village” as the Villages-in-the-city phenomenon

Since the 2010s, an increasing number of publications have associated the term *urban village* with the context of rapid urbanisation, particularly in China and other countries of the Global South. In this context, the term refers to the phenomenon of ViCs – traditional rural settlements engulfed by urban expansion [16–21]. Studies on ViCs have gained significant prominence and now represent the dominant strand in *urban village* scholarship [1].

While ViCs are most commonly studied in China (*chengzhongcun* in Chinese), they are also found in a variety of Global South contexts [1,22], including Vietnam [22–25], India [26–29], Indonesia [30–33], Malaysia [34,35]. In Indonesia, Malaysia, and Singapore, these settlements are often referred to as *urban kampung* [32,36,37].

A defining feature of ViCs is their physical encirclement by expanding urban areas. They frequently serve as hubs for rural-to-urban migrants, leading to extremely high population density, overstretched infrastructure, and ambiguous land tenure. Spontaneous development and unauthorised construction – often in the absence of effective planning oversight – have led many scholars to categorise ViCs as a form of “informal settlement”.

However, recent studies have also highlighted the positive attributes of ViCs. These include walkability, accessibility, mixed land use, community cohesion, and a high degree of self-governance – characteristics aligned with the *Urban Village Concept* [16]. Also, ViCs contribute significantly to urban transitions: they provide alternative income sources for villagers displaced from agriculture, offer affordable housing for low-income migrants, ease public welfare burdens, and supply labor to the urban economy [16,38,39]. Proximity to economic and civic centres grants residents better access to opportunities, distinguishing ViCs from other informal settlements worldwide [40]. Moreover, their compactness and strong social networks encourage non-motorised mobility and attract creative communities, contributing to the growth of local creative industries [41,42].

### Towards a synthetic perspective on urban villages

Despite sharing the same label, the three strands – *ethnic enclaves*, the *Urban Village Concept*, and *ViCs* – have largely developed in parallel. *Ethnic enclave* studies focus on identity and migration; *planning-oriented* accounts privilege normative design models; and *ViCs* research foregrounds informality, hybridity, and contested redevelopment. Few studies have examined how these perspectives might intersect within a single empirical context.

As a result, the conceptual potential of the *urban village* remains fragmented. A synthetic perspective offers an opportunity to examine how community, identity, informality, and planning ideals coexist and interact in practice. Such an approach is particularly relevant for cities in the Global South, where imported planning models encounter historically layered settlement patterns and socio-cultural hybridity.

## Relevance to the case study

Although the *urban village* has been theorised through diverse lenses, empirical settings in which these interpretations can be examined simultaneously remain rare. Da Lat and its surrounding areas offer such a setting, where *long-established migrant settlements*, *indigenous villages*, and a *state-led urban village planning model* coexist within a shared urban development boundary. This convergence makes Da Lat particularly suitable for examining the coexistence and interaction of different *urban village* forms.

### Da Lat city and its vicinity

The spatial scope of this study follows *Plan No. 704*, encompassing Da Lat city and Lac Duong, Don Duong, Duc Trong and (part of) Lam Ha districts. The area encompasses approximately 335,930 ha, with a projected total population of 700,000–750,000 by 2030.

Da Lat presents a distinctive case of urban formation shaped by colonial planning and successive waves of domestic migration. Initially identified in 1893 as an uninhabited highland area, the city was developed by the French colonial administration as a hill station intended to embody European urban ideals. This colonial core emerged as an “island” within a broader region historically inhabited by indigenous communities. Subsequent migration waves from northern and central Viet Nam gradually populated both the city and its surrounding districts, producing a complex socio-spatial landscape characterised by overlapping settlement histories and ethnic diversity. [43–51]

### The Green Urban Village pilot project

Despite the absence of an official national definition of the *urban village*, Lam Dong became the first locality to propose a *Green Urban Village* model aligned with the strategic orientation of *Plan No. 704*. Within the context of Da Lat’s expansion towards a satellite urban system, aiming by 2050 to establish newly high-quality residential areas [52], the model is supported by *Decision No. 1528/QĐ-TTg* issued by the Prime Minister in 2015, which provides a number of specific mechanisms and policies including authorisation for its pilot implementation. In 2016, Lam Dong Provincial Department of Construction defined the terms as follow [53]:

Interspersed among urban areas are zones for the development of high-tech agricultural production combined with residential areas - these are *urban villages*, offering a quality of life comparable to that of the city, and developed according to green and sustainable principles - referred to as *Green Urban Villages*.

**Fig 1:**
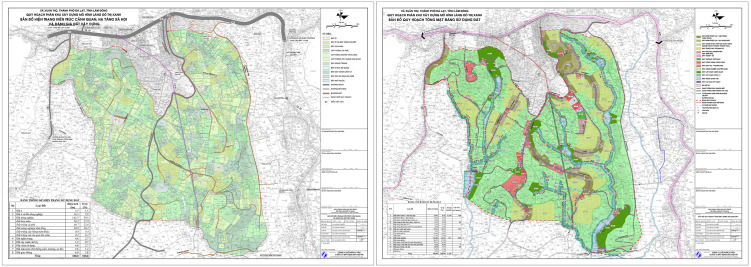
The existing condition and the master plan of the pilot Green Urban Village. Source: Reprinted from [54] under a CC BY license, with permission from VITILAND, original copyright 2026.

Approved in 2021, the pilot project was designated in Xuan Tho commune (Fig 1) [54]. The selected site’s existing condition consists mostly of agricultural land (79.3%) with a sparse population of approximately 680 people. Notably, no pre-existing village settlement exists within the project boundary. Remaining largely vacant until now, this greenfield orientation reveals a significant gap between policy ambition and socio-spatial reality.

While drawing inspiration from the *Garden City* movement, *New Urbanism*, and the *Urban Village Concept* [52,53,55], the model exhibits several conceptual limitations. It prioritises low-density, single-use residential development and lacks clear urban design criteria, contradicting the mixed-use, multifunctional principles central to its reference models. More critically, the absence of existing social structures and cultural continuity raises questions about the authenticity of the “village” label and the long-term sustainability of the model.

### A historical stream with the companionship of village communities and ethnic clusters

Understanding the historical formation of Da Lat and its surrounding districts requires tracing the intertwined processes of colonial planning, successive waves of domestic migration, and the emergence and enduring presence of village communities. These processes not only shaped the city’s demographic and spatial structure but also fostered conditions for the emergence of ethnic or localised place-of-origin clustering. This section provides a historical account of these dynamics, highlighting how villages were embedded within the urban fabric of Da Lat and how patterns of settlement in the vicinity resonate with characteristics commonly associated with *ethnic enclaves*.

During the early phases of Da Lat’s construction, the French colonial administration prioritised transportation infrastructure, relying heavily on Vietnamese labourers who had been forcibly relocated from the northern and central regions of Viet Nam [49]. From the original intention of a “French resort station”, by 1919, the growing Vietnamese population prompted French planners to designate specific areas for them within their master plans [48,50]. Then, the 1930s saw a surge in population following the completion of transport networks, witnessing the emergence of a “little Vietnam” within Da Lat. Between 1946 and 1953, nearly a dozen villages were founded, often based on the regional origins of incoming migrants [50].

Derived from the city’s historical gazetteer [48], Vietnamese migrants from the northern and north-central provinces formed a substantial portion of Da Lat’s early population. After 1954, the Geneva Accords and the dissolution of the Montagnard Autonomous Region (1955) spurred further migration from the North, including ethnic minorities, reshaping Da Lat’s demographics. By then, the city comprised 37 villages. After 1975, the city continued to absorb various new populations under the state’s new economic zones policy.

Scholars [48–50,56,57] affirm that, throughout history, Da Lat’s villages were initially settled by migrants from northern and central Vietnam. The author’s previous research [58] argues that Da Lat developed as a city in parallel with the existence of villages embedded within its urban fabric. Also, the study emphasised their distinct residential origins and settlement histories. In particular, residents often clustered according to shared places of origin. A notable example is Ha Dong flower village, where the majority of inhabitants came from Ha Dong (now part of Hanoi). Today, this village has been officially recognised as a “traditional craft village” located within the heart of the city. The presence of village temples, collective activities such as worship ceremonies, and, especially, agricultural landscape within urban boundaries suggest that certain rural village characteristics were preserved in the city's development. These features form the basis for the hypothesis regarding the existence of ViCs in Da Lat.

The districts surrounding the city share a common demographic characteristic: a diverse and mixed population in terms of ethnicity and origin. These areas are home to both indigenous groups, ethnic minority migrants from northern provinces, and Kinh settlers. Migration to these areas occurred in three major waves [43,45–47]. The first wave began with the colonial administration’s decision to develop Da Lat as a hill station. Kinh migrants were brought to Lam Dong to construct transportation infrastructure, build settlements, and establish plantations for tea, coffee, and vegetables. The second major migration occurred between 1954 and 1975. From 1954 to 1955, following the signing of the Geneva Accords, ethnic minorities from northern Vietnam – mainly the Thai, Tay, Nung, and Hoa ethnic communities – along with Catholic followers and relatives of French veterans, were forcibly resettled in the South. From 1955 to 1975, political repression by the Republic of Vietnam (South Vietnam) led to the migration of tens of thousands of people from coastal provinces in Central Vietnam to Lam Dong. The third wave began in 1975 and has progressed rapidly. It had been driven largely by national policies aimed at redistributing labor and population to promote socio-economic development in the Central Highlands and Southeastern regions. This demographic growth had been fueled by both planned migration programs and spontaneous migration.

Although these migration dynamics do not stem from international migration flows – as is often the case in studies on *ethnic enclaves* – certain areas in the vicinity still display characteristics of ethnic clustering, where minority communities form localised concentrations due to internal migration patterns.

### Original socio-spatial structures of Vietnamese Kinh villages

In Vietnam, the village has traditionally been regarded as a “miniature nation”, embodying the essential structures of society [59]. The Northern Delta is recognised as the cradle of Vietnamese Kinh’s village culture – the Kinh being the majority ethnic group of the country [60–62]. From the fifteenth century onward, as the central coastal regions were incorporated into the national territory, Kinh villages in the Central provinces gradually took shape there as well [61]. With the southward expansion, Kinh villages in the Mekong Delta emerged much later, with a history of only about three centuries [63]. Given that the villages of Da Lat were initially settled by migrants from northern and central Vietnam, this study confines its scope to these two regional traditions.

Over centuries, northern villages developed highly cohesive and resilient organisational structures, governed in parallel by formal administrative apparatuses and autonomous self-governance. By contrast, central villages emerged through the southward expansion of the nation. Unlike the northern model, characterised by clearly demarcated boundaries between villages, kinship groups, neighborhoods, and households – reinforced by symbolic community landmarks such as communal houses, temples, pagodas, banyan trees, and village wells – the social and spatial markers of cohesion in central villages have been comparatively less pronounced [64]. However, in central villages, kinship plays a pivotal role in social life. Lineages credited with land reclamation and village founding are especially revered, with rituals and festivals centered on these ancestral figures [61].

In both northern and central Vietnam, the communal house (đình làng) stands as the most significant architectural and spiritual landmark, embodying the village’s cultural identity. Among folk beliefs, the cult of the village deity (Thành Hoàng) most fully reflects collective consciousness, while the village festival has long served as a vital arena for solidarity, cultural transmission, and creativity [64–67]. Notably, elements of these traditions remain, more or less, present within Da Lat’s settlements.

In sum, the Vietnamese Kinh village can be understood as a relatively self-contained socio-spatial unit, encompassing public facilities, subsistence-based economic activities, and a distinct “spiritual universe” governed by customs and village regulations. Despite historical and institutional transformations, enduring structures such as kinship networks and traditional norms have proven resilient. Consequently, when migrants from northern and central Vietnam settled in Da Lat and its surroundings, these *social*, *economic*, *environmental*, and *governance* features of the village were reproduced – though in new forms – within the emerging settlements.

### Indigenous villages in urban areas of the Central Highlands

This section builds upon the foundational work of Nguyen Hong Ha [68]. Indigenous villages in the Central Highlands exhibit distinctive socio-spatial structures that persist despite increasing urban integration. While many villages were established prior to the French colonial period, others were resettled or reshaped under colonial and wartime interventions. Their built environment reflects both continuity and transformation: traditional stilt houses, longhouses, and communal houses (nhà rông) coexist with newer ground-level dwellings influenced by Kinh settlement practices. Settlement patterns differ across ethnolinguistic groups – Austronesian communities such as the Ê Đê and Jrai often occupy dispersed plots of 20–40 hectares with defined household compounds, while Mon-Khmer groups like the Bahnar and Xơ Đăng reside in compact, nucleated clusters averaging 10–15 hectares, with houses closely arranged and public spaces interlinked by footpaths.

Cultural and spiritual landmarks remain central. The communal house, ancestral houses of village founders, ritual spaces, water sources (giọt nước), cemeteries, and gong culture arenas function as symbolic and functional anchors of collective life. Intangible heritage – including epic traditions, gong music, festivals, and ritual practices – continues to structure social cohesion. Kinship retains a pivotal role, with the authority of village elders and customary laws governing land use, inheritance, and dispute resolution.

Economically, some villages have adapted by engaging in small-scale tourism and cultural performances, while others face challenges such as land commodification, Kinh in-migration, and uneven urban planning oversight. Public infrastructure and spatial planning often remain limited to preserving existing forms rather than guiding future development. Ecologically, villages sustain a close relationship with the natural environment through swidden agriculture, household garden-pond-livestock systems (VAC), and cyclical resource management, which together form resilient socio-ecological systems.

These structures illustrate how indigenous villages in the Central Highlands function not only as residential units but also as socio-spatial systems that intertwine cultural identity, governance, ecology, and adaptation within expanding urban contexts.

## Data and methodology

### Step 1 – Constructing spatial data and locating the hypothesised urban villages

Based on the historical review, the 1960s were identified as a period of relative stability in the spatial structure of villages in Da Lat and its vicinity. Accordingly, historical maps produced between 1964 and 1971 [69] were used as the primary spatial references. Villages were georeferenced and digitised as point features, then cross-validated and supplemented using documentary sources [48–51,54,56,70]. This process also enabled the inclusion of nine *traditional craft villages* and three *craft villages* as officially recognised in 2023.

Villages located within the planned *urban development boundary* were hypothesised as potential *urban villages*. To spatially contextualise these locations, the current *commune-level administrative boundary* layer was overlaid with the *urban development boundary* defined in *Plan No. 704*. A *500-meter buffer* was applied to each village point to account for positional uncertainty in historical maps and manual digitisation. This distance, corresponding to a 5–7 minute walking radius commonly used in neighbourhood-scale urban studies, was intended to minimise false exclusion rather than delineate precise village boundaries. Villages whose buffer intersected the *urban development boundary* were classified as *urban villages*.

Verification interviews were conducted with commune and ward leaders in all relevant administrative units to confirm the continued existence and administrative recognition of identified villages A semi-structured checklist covered village name, establishment period, location, area, population size, and population origins. Interview data were used solely for validation and not subjected to qualitative analysis.

### Step 2 – Constructing observational data from literature review

This step aimed to identify variables for defining *urban villages* in Da Lat and its vicinity. Relevant literature was retrieved from Scopus and ScienceDirect using the search terms “urban village”, “ethnic enclaves”, “Urban Village Concept”, “villages in the city”, and “ViCs”. Articles were selected based on three criteria: (i) peer-reviewed status, (ii) explicit engagement with *urban villages*, *ethnic enclaves*, or *ViCs*, and (iii) relevance to socio-spatial characteristics.

Variables were extracted from definitions, conceptual discussions, and empirical descriptions. Duplicate or overlapping variables were consolidated through semantic comparison, merging indicators that described the same underlying phenomenon at a higher level of abstraction.

To reflect settlement origins specific to the case study, additional literature on *traditional villages in northern and central Viet Nam* and on *indigenous villages in urban areas of the Central Highlands* was incorporated. Sources on these Vietnamese Kinh villages were drawn from domestic scholarly works [61,64–67,71–75], while insights on indigenous villages followed established ethnographic research of Ha [68].

In total, 91 articles were reviewed (Table 1), yielding 432 variables, including 267 derived from the three major interpretations of the *urban village* and 165 specific to the Da Lat context. Variables were synthesised through theory-informed qualitative content analysis conducted by the author as a single primary coder to ensure conceptual consistency. Coding combined deductive guidance from the theoretical framework with inductive refinement based on recurring themes.

**Table 1:** Count of articles, context and initial variables.

Literature topic	Context	Count of articles	Count of variables	
			Definition-based	Local context-based
Ethnic enclave	Worldwide	4	11	
Indigenous villages in urban areas – Central Highlands VN	Indigenous villages in urban areas – Central Highlands VN	1		76
Traditional villages – Central VN	Traditional villages – Central VN	1		6
Traditional villages – Northern & Central VN	Traditional villages – Northern & Central VN	8		74
	Traditional villages – Northern VN	1		5
Traditional villages – Northern VN	Traditional villages – Northern VN	1		9
Urban village concept	Da Lat city	2	16	
	UK	3	17	
	UK – America	5	56	
Village in the city	China	52	110	
	China, India	3	20	
	China, Viet Nam	1	15	
	India	1	18	
	Indonesia	4	29	
	Malaysia	2	13	
	Viet Nam	3	32	
*Total (raw)*		*92*	*337*	*170*
**Total (duplication removed)**		**91**	**267**	**165**

All variables were subsequently organised into four thematic categories – *social, economic, environmental*, and *institutional & governance* – reflecting a commonly adopted analytical structure in sustainable development research aligned with the Environmental, Social, and Governance (ESG) framework, while explicitly incorporating the Economic dimension.

### Step 3 – Defining observed variables

#### Variables assessment using Modified Delphi method.

**Fig 2:**
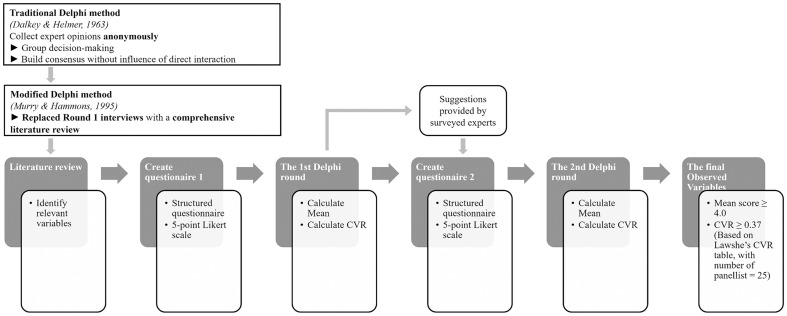
Defining OVs by Modified Delphi Method. Source: author.

Preliminary observed variables (OVs) were defined using a Modified Delphi method [76] (Fig 2), replacing initial interviews with variables derived from the literature review.

In Round 1, experts assessed each variable on a 5-point Likert scale according to its relevance for identifying *urban villages* in Da Lat and its vicinity. Mean scores and Content Validity Ratio (CVR) were calculated. Variables with Mean ≥ 4.0 were retained, those with Mean < 3.5 or low CVR were excluded; and mid-range items (Mean 3.5–3.99), together with newly suggested variables, proceeded to Round 2.

Due to time constraints, a third Delphi round was not conducted. This limitation was partially mitigated by combining expert assessment with data-driven topic modelling and subsequent multi-criteria weighting.

Experts were selected through purposive and snowball sampling and included researchers, planners, architects, and local administrators with professional experience in Da Lat. Familiarity with the *Green Urban Village* was required to ensure informed judgement, while diversity in institutional background was maintained to reduce normative bias. Following standard Delphi practice, 25 experts were engaged, and a minimum CVR threshold of 0.37 was applied based on Lawshe’s table.

#### Criteria modeling using Latent Dirichlet Allocation algorithm.

After Round 1, the temporary OVs set – including retained variables, mid-range items, and newly suggested variables – was grouped into thematic criteria using Latent Dirichlet Allocation (LDA). LDA is a machine learning-based topic modeling technique that identifies latent thematic structures in textual data by analysing word co-occurrence patterns. As a generative probabilistic model, it assumes that documents are composed of multiple latent topics, each represented by a distribution of words, enabling a systematic and data-driven extraction of underlying themes [77].

LDA was applied as an exploratory clustering tool to structure a large and heterogeneous variable set, rather than as a substitute for expert consensus. Its use was motivated by practical constraints related to expert availability and project timeline, and it served to support subsequent evaluation and weighting procedures.

The analysis was conducted in a Python environment using open-source libraries. *Gensim (v4.3.3)* was used to train the LDA model, with text preprocessing performed using *NLTK*, including stopword removal, tokenisation, and lemmatisation. Model training employed controlled random initialisation (random_state = 42) to ensure reproducibility, with iterative updates (update_every = 1), a chunksize of 100 documents, and 10 passes through the corpus to support stable topic convergence. The Dirichlet prior for topic distribution (alpha) was set to auto, allowing the model to infer an appropriate asymmetric prior from the data, while other hyperparameters were kept at default settings.

**Fig 3:**
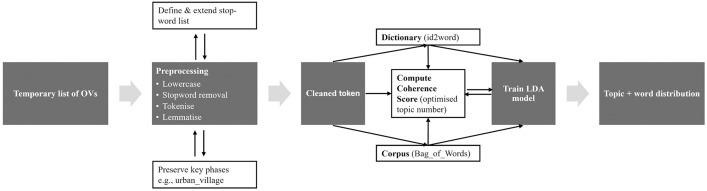
The process of topic modeling using LDA. Source: author.

Model validation prioritised semantic interpretability over predictive performance. The number of topics was optimised by comparing coherence scores across models with different topic counts, enabling the selection of semantically coherent and interpretable topic structures. Perplexity scores were examined during model iteration but were not used as the primary selection criterion, given their limited interpretability for small, concept-driven textual datasets. An overview of the modelling workflow is presented in Fig 3.

Each topic was characterised by its ten highest-weighted keywords and consolidated into final evaluation criteria through theory-informed interpretation. To mitigate potential researcher bias, the resulting criteria were further validated through expert-based weighting using the Analytic Hierarchy Process (AHP).

#### Calculating criteria weight with the Analytic Hierarchy Process.

AHP [78] was applied to derive criterion weights based on expert *pairwise comparisons* using a *9-point scale*, ranging from “equal importance” to “extreme importance”. The subsequent steps involved constructing the pairwise comparison matrix, normalising, calculating the weights, and assessing the consistency of responses via *Consistency Index* (CI) and *Consistency Ratio* (CR). The result would be a ranked list of topics based on calculated weights. However, when inconsistency arises in the matrix, repeating interviews is unfeasible due to time constraints. Instead, the matrix was refined through a multi-objective optimisation algorithm, explained in the following step.

#### Addressing inconsistencies in AHP matrices using Evolutionary Multi-objective Optimisation (EMO).

**Fig 4:**
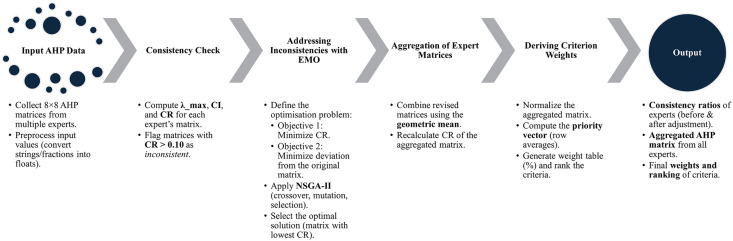
Workflow of AHP consistency check and weight derivation. Source: Author.

In the AHP, inconsistency in expert judgments arises when the CR exceeds the acceptable threshold of 0.1, indicating that the pairwise comparisons may be unreliable. This often results from the cognitive difficulty of evaluating numerous items simultaneously, leading to partial or unacceptable consistency. To improve this, in the *Python environment,* EMO method via *Non-dominated Sorting Genetic Algorithm II* (NSGA-II) [79] was selected for its efficiency, robustness, and ability to optimise CR while preserving the original matrix structure, making it ideal for small samples. A summarisation of the process can be found in Fig 4.

### Step 4 – Scoring areas hypothesised as urban villages

A field survey was conducted using the finalised OVs checklist. Collaborators were recruited from civil servants working in wards and communes encompassing the studied villages and were identified in consultation with local administrations based on their familiarity with settlement histories, land use, and community conditions. Each village was assessed by five collaborators, allowing triangulation across informants while maintaining feasibility. The number of collaborators was selected to balance diversity of local perspectives with practical constraints, prioritising cross-validation of observations over statistical representativeness. Responses were recorded in binary form (yes/no) to enhance inter-rater consistency.

Drawing on literature, the finalised OVs, field observations, and collaborator input, settlements were found to be demographically mixed rather than homogeneous. Accordingly, a context-based evaluation framework was applied (Table 2), assigning different sets of OVs to different settlement types to avoid normatively biased comparisons.

**Table 2:** Scoring framework.

Site context ▷	Villages with migrants from Northern and/or Central Vietnam	Indigenous villages	Indigenous villages mixed with migrants	The piloted Green Urban Village
Literature topic ▽				
Ethnic enclave	●	●	●	●
Village in the city	●	●	●	●
Urban village concept				●
Indigenous villages in urban areas – Central Highlands VN		●	●	●
Traditional villages – Northern & Central VN	●		●	●
Traditional villages – Northern VN	●		●	●

For the unimplemented *Green Urban Village* pilot project, assessment was conducted by the author based on site observation and analysis of official planning documents [54]. The project was first evaluated using *Urban Village Concept* OVs, followed by the full OVs set for comparability.

Missing or invalid responses were excluded. For each OV, an average score was calculated across valid responses, and a simple majority rule was applied (true > 0.5; false < 0.5). Ambiguous results (average = 0.5 or blank) were excluded from further analysis. While transparent and consistent, the absence of sensitivity analysis is acknowledged as a limitation.

To evaluate each *hypothesised urban village*, a criterion-based scoring procedure was applied using the finalised OVs and criterion weights derived from the EMO-optimised AHP analysis. For each village, only categories relevant to its settlement context were considered, ensuring that villages were assessed against variables applicable to their socio-spatial characteristics rather than against the full variable set indiscriminately.

For each evaluation criterion, two quantities were calculated:

- The number of matched OVs, defined as the count of unique OVs associated with that criterion that were positively identified in the village; and
- The total number of applicable OVs, defined as the count of unique OVs associated with the same criterion and belonging to categories relevant to the village.

The criterion-specific score for village *v* under criterion *c* was calculated as:


$$Score_{v,c}=\left(\frac{N_{Matched}}{N_{Total}}\right)\times W_{c}$$

where *Nmatched* represents the number of OVs identified in village v for criterion c, Ntotal represents the total number of applicable OVs for that criterion, and *Wc* denotes the weight of criterion *c*. If a criterion had no applicable OVs or no identified variables, the score for that criterion was recorded as missing and excluded from aggregation.

Criterion-level scores were subsequently aggregated to produce an overall score for each village. These overall scores are relative indices, intended to support comparison across villages within the study area rather than to represent absolute measures of performance or predefined thresholds of “good” or “poor”. Interpretation therefore focuses on comparative patterns, internal ranking, and differences across village types, rather than on normative cut-off values.

Variables derived from the *Urban Village Concept* were not applied to existing villages, as they represent prescriptive design principles rather than empirically observable features. Applying these variables retroactively would risk introducing normative bias into the assessment. The binary scoring scheme simplifies complex socio-spatial phenomena but was intentionally used to reduce subjectivity among non-academic collaborators and to support systematic cross-site comparison; this trade-off is acknowledged as a limitation.

Given the exploratory and case-based nature of the study, inferential statistics and confidence intervals were not applied. Robustness was instead sought through triangulation, context-sensitive variable selection, and transparent aggregation rules.

### Ethics statement

This study involved expert consultations, verification interviews with local officials, and community-based assessments conducted by civil servants at the ward and commune levels. All participants were informed of the study’s purpose, the voluntary nature of participation, and their right to withdraw at any time without consequences.

Informed consent was obtained verbally from all participants prior to data collection. Verbal consent was appropriate given the non-sensitive nature of the study and the professional roles of participants. Consent was documented through field notes and survey records.

Formal ethical approval was not required, as the study did not involve vulnerable populations, sensitive personal data, or experimental interventions.

## Result

### The hypothesised urban villages

**Fig 5:**
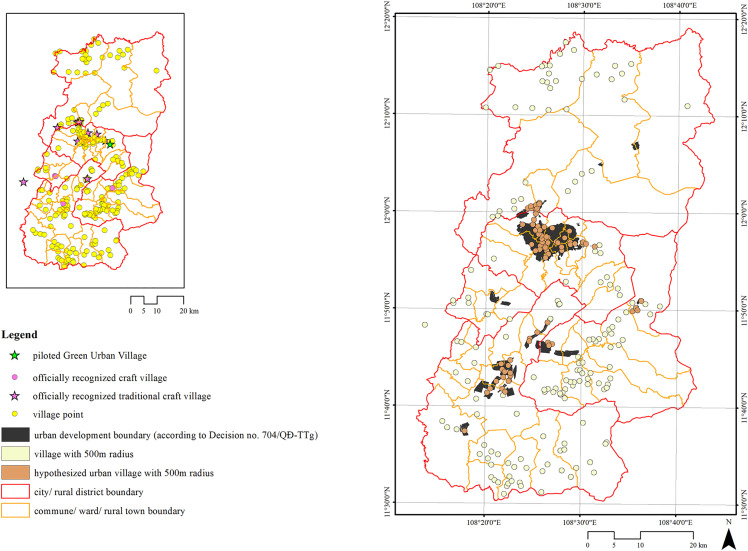
Positioning the hypothesised urban villages. Source: by author, with the commune-level administrative boundary layer and the urban development boundary layer extracted and adapted from the Land use plan for Da Lat city and its vicinity to 2030, under a CC BY license, with permission from Department of Construction of Lam Dong province, original copyright 2026.

Alongside the vacant site designated for the *Green Urban Village* pilot project, 75 verifiable villages were identified within the planned *urban development boundary* and hypothesised as *urban villages* (Fig 5). Based on documentary sources and verification interviews (Table 3) nearly all villages in Da Lat originated from migrant populations from northern and central Viet Nam, whereas indigenous settlements are more prevalent in Don Duong and Duc Trong districts. All *hypothesised urban villages* in Lac Duong are of indigenous origin, although several – including Bac Hoi, Drong, and Kekaff – exhibit mixed demographic compositions. No villages were identified within the *urban development boundary* of Lam Ha District.

**Table 3:** List of the hypothesised urban villages.

		Mapping						
						Literature review		
						Interview		
No.	Village	Longitude	Latitude	City/District	Ward/Commune	Origin of residents		
						Northern Vietnam	Central Vietnam	Indigenous groups
1	An Hoa	108.434050	11.942444	Da Lat	Ward 1		●	
2	Thien Thanh	108.432089	11.940177			●	●	
3	Anh Sang	108.435937	11.939552				●	
4	Da Loi	108.464644	11.934735		Ward 10		●	
5	Hong Lac	108.464261	11.940617			●	●	
6	Nam Ho	108.479016	11.949190		Ward 11		●	
7	Da Phuoc	108.501026	11.945875				●	
8	Tu Tao	108.501248	11.949243			●	●	
9	Tay Ho	108.479436	11.945030				●	
10	Sao Nam	108.487479	11.946688				●	
11	Thai Phien traditional craft village of flower	108.477682	11.968276		Ward 12		●	
12	My Loc	108.433633	11.953909		Ward 2	●	●	
13	Vo Tanh	108.440357	11.947929			●	●	
14	Xuan An	108.438114	11.934042		Ward 3		●	
15	Tan Lac	108.441466	11.928518				●	
16	Saint Jean	108.436368	11.922178			●		
17	Nam Thien	108.425865	11.936037		Ward 4	●	●	
18	My Thanh	108.426915	11.942221		Ward 5	●	●	
19	Du Sinh	108.418859	11.933997			●		
20	Van Thanh traditional craft village of flower	108.412032	11.944806			●	●	
21	Da Trung	108.429332	11.954579		Ward 6	●	●	
22	Da Cat	108.430715	11.948846			●	●	
23	Da Thuan	108.429702	11.954488			●	●	
24	Da Phu	108.413760	11.978660		Ward 7	●	●	
–	Cuu Binh Si	108.415583	11.987217				disappeared	
25	Mang Line	108.395770	11.978753					●
26	Da Thanh	108.425596	11.963018				●	
27	Cao Ba Quat	108.428807	11.960227			●	●	
28	Cao Thang	108.424037	11.962317			●	●	
29	Phuoc Thanh	108.418453	11.997668			●	●	
30	Kim Thach	108.418329	11.969035			●	●	
31	Tung Lam	108.422773	11.975783			●	●	
32	Nguyen Sieu	108.426221	11.969053				●	
33	Thanh Mau	108.433544	11.974635				●	
34	Nghe Tinh	108.440364	11.957463		Ward 8		●	
35	Da Thien traditional craft village of flower	108.447700	11.973317				●	
36	Ha Dong traditional craft village of flower	108.437750	11.958320			●		
37	Co Giang	108.457580	11.947976		Ward 9		●	
38	Chi Lang	108.469349	11.956465			●	●	
39	Da Loc	108.521741	11.941939		Xuan Tho		●	
40	Xuan Thanh traditional craft village of flower	108.506608	11.946485				●	
41	Quang Lac	108.603806	11.849267	Don Duong	D'Ran	●	●	
42	Tan Lap	108.588876	11.831731		Lac Lam	●	●	
43	Lac Thien	108.594527	11.837116		D'Ran	●	●	
44	Lac Quang	108.597343	11.833158			●	●	
45	Bac Hoi	108.441683	11.776615		Da Ron	●	●	●
46	Drong	108.449116	11.773892			●	●	●
47	Kekaff	108.443564	11.771438			●	●	●
48	Klong A	108.440801	11.810927	Duc Trong	Hiep An			●
49	Quang Hiep	108.423084	11.793950		Hiep Thanh		●	
50	Finom	108.410128	11.783157			●	●	
51	Phu Thanh	108.409189	11.778537			●	●	
52	Nghia Lam	108.360766	11.739345		Lien Hiep	●	●	
53	Residential Group 5 craft village of mushroom growing	108.362697	11.729956		Lien Nghia	●	●	
54	Lien Hiep	108.376578	11.745943			●	●	
55	Cao-Thai -Son	108.373192	11.736645			●		
56	Cao-Bac-Lang	108.377035	11.719371			●		
57	Thai	108.376716	11.723853			●		
58	Nam Son 3	108.352574	11.709468			●	●	
59	Nam Son 1	108.364646	11.716744			●	●	
60	Nam Son 2	108.367082	11.725720			●	●	
61	Dang Sorn	108.298205	11.623837		Ninh Gia			●
62	Luc Nam	108.374836	11.711231		Lien Nghia	●	●	
63	Phu Hoi 2	108.373968	11.709733		Phu Hoi	●	●	
64	Phu Hoi 1	108.363079	11.696544			●	●	
65	Tchirong Tambor	108.342017	11.696803					●
66	Tchirong Kra	108.336461	11.689700					●
67	Pre Rion	108.368069	11.691476					●
68	Traditional craft village of brocade weaving	108.413413	12.009598	Lac Duong	Lac Duong			●
69	Bon Langbiang traditional craft village of wine making	108.418481	12.011918					●
70	B'Neur 4	108.421309	12.002861					●
71	B'Neur 3	108.420468	12.006035					●
72	Bon Deung	108.424917	12.016375					●
73	Dangia	108.424422	12.014116					●
–	Da Phu	108.404542	12.006721		Lat		disappeared	
74	B'Neur 2	108.410752	12.006995		Lac Duong			●
75	B'Neur 1	108.409842	12.010249					●

### Criteria and observed variables

#### Results from the first Delphi round and AHP.

**Fig 6:**
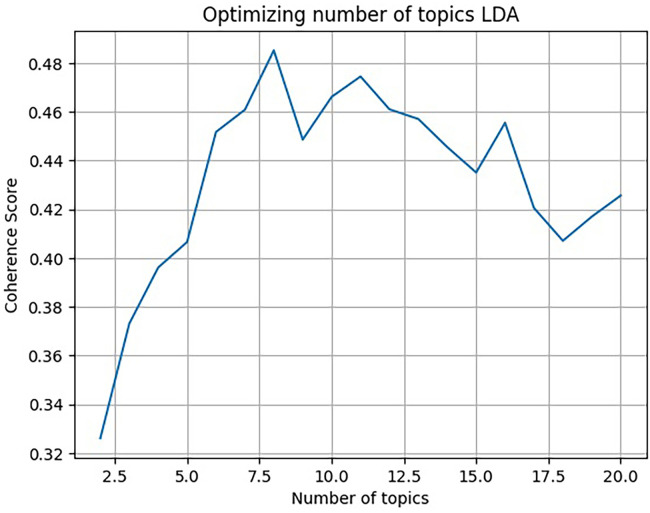
Optimised number of topics via LDA. Source: author.

From an initial pool of 432 variables, the first Delphi round retained 114 variables, forwarded 100 for reconsideration, and incorporated 46 newly proposed variables. Using coherence-based optimisation, all variables were grouped through LDA into eight thematic topics, subsequently consolidated into eight *Evaluation Criteria* (Fig 6; Table 4).

**Table 4:** Eight criteria to define urban villages in Da Lat city and its vicinity.

No.	Top 10 words with weights	Criterion	Weight
1	cultural (0.0837), value (0.0621), heritage (0.0402), intangible (0.0391), traditional (0.0287), culture (0.0184), festival (0.0182), village (0.0180), highland (0.0174), folk (0.0174)	**1. Cultural Preservation:** Urban villages preserve *cultural value* through traditional *culture*, *festivals*, and *folk* practices. In Vietnam’s Central *Highlands*, these *villages* reflect *intangible heritage* rooted in local traditions, strengthening cultural identity and connecting the community to its past.	15.62
2	village (0.0844), management (0.0343), agricultural (0.0224), integrating (0.0186), member (0.0184), product (0.0143), hamlet (0.0141), road (0.0141), alley (0.0138), relation (0.0131)	**2. Social and Spatial Cohesion** Urban villages often retain *village*-like structures, with *management* practices shaped by *agricultural* traditions. The *integration* of community *members*, local *products*, and physical elements such as *roads* and *alleys* helps maintain social *relations* and ensures spatial coherence within the *village*.	14.12
3	area (0.0840), planning (0.0290), urban (0.0284), production (0.0243), green (0.0196), residential (0.0190), village (0.0190), urban_village (0.0178), land (0.0178), agricultural (0.0178)	**3. Urban and Agricultural Integration** *Urban village areas* integrate *residential* and *agricultural land*, with *planning* approaches that prioritise *green* spaces and support both urban and agricultural *production*. These areas reflect the blending of *urban* development with traditional *village* forms, fostering a balanced relationship between development and *agriculture*.	13.65
4	community (0.0791), village (0.0390), social (0.0360), space (0.0276), life (0.0270), house (0.0245), central (0.0219), resident (0.0210), culture (0.0170), communal (0.0151)	**4. Community and Social Structures** Urban villages foster a sense of *community* by providing *social spaces* and supporting *communal life*. These spaces are often centred around *community houses* and shared *cultural* practices, enabling *resident* interaction and reflecting the *central* role of traditional village-based *social* structures.	12.44
5	urban (0.0475), resident (0.0269), people (0.0268), need (0.0250), village (0.0229), building (0.0211), population (0.0200), activity (0.0156), maintaining (0.0151), whole (0.0146)	**5. Addressing Resident Needs** Urban villages address the *needs* of *residents* by providing both *urban* living quality and *maintaining village activities*. As the *population* increases, the demand for housing grows, leading to higher *building* density to meet these needs.	12.32
6	urban (0.0525), village (0.0453), group (0.0254), construction (0.0239), migrant (0.0233), process (0.0173), ethnic (0.0171), tradition (0.0163), among (0.0139), housing (0.0110)	**6. Migrant and Ethnic Integration** Urban villages emerge through *processes* of *housing construction* and development, often involving *migrant* or *ethnic groups*. These *villages* illustrate the interaction between *urban* growth and *traditional* practices, contributing to the cultural and social fabric of the broader *urban* environment.	12.21
7	urban (0.0308), economic (0.0261), sustainable (0.0240), type (0.0203), ensuring (0.0187), house (0.0178), garden (0.0173), infrastructure (0.0166), development (0.0135), positive (0.0126)	**7. Sustainable Economic Development** Urban villages promote a *sustainable economy* by integrating *urban* agriculture, *garden* spaces, and *housing* into *development* plans. This approach supports both local *economic* growth and the long-term environmental *sustainability* of the community.	11.93
8	natural (0.0336), identity (0.0308), village (0.0236), important (0.0209), close (0.0188), living (0.0174), environment (0.0172), role (0.0167), preserve (0.0159), use (0.0152)	**8. Environmental and Ecological Integrity** The *natural* environment plays a *key role* in defining the *identity* of urban villages. *Preserving natural* landscapes and ecological systems is *essential* for maintaining both the cultural integrity of the *village* and its long-term *environmental* health.	7.71

When these criteria were weighted using AHP, the aggregated comparison matrix exhibited substantial inconsistency (CR = 0.49), with 19 of 25 experts exceeding the acceptable threshold. This reflects the cognitive difficulty of pairwise comparison across abstract and interdisciplinary criteria among experts with heterogeneous backgrounds. Rather than discarding expert input, EMO optimisation was applied to reduce inconsistency while preserving the overall structure of judgments. The resulting weights indicate a balanced prioritisation, with no criterion disproportionately dominant or marginalised (Table 4).

#### Finalised observed variables.

Following the second Delphi round, 205 OVs were finalised (S1 Table). Variables associated with the *Urban Village Concept* recorded the highest retention rate, suggesting an expert inclination toward future-oriented planning models. Although *ViCs*-related variables experienced the highest rejection rate, nearly 50 were retained, underscoring the continued analytical relevance of this phenomenon.

Only three variables related to *ethnic enclaves* were retained. While limited in absolute terms, this represents 27% of the original *ethnic enclave* pool (11 variables across four core studies). Importantly, these retained variables capture foundational dimensions of *ethnic enclaves*: (i) adaptation to the urban environment, (ii) tightly knit community structures, and (iii) conscious maintenance of identity. These dimensions align respectively with the criteria of *Social and Spatial Cohesion*, *Community and Social Structures*, and *Cultural Preservation*, indicating that *ethnic enclave* characteristics persist as latent, cross-cutting attributes rather than as a dominant or standalone settlement form.

Context-based village topics further reveal that most variables specific to *Traditional villages – Northern Vietnam* or *Traditional villages – Central Vietnam* were rejected in isolation, while hybridised features were retained. *Indigenous villages in urban areas of the Central Highlands* retained a comparatively larger number of variables. Overall, literature strands related to the *Urban Village Concept*, *ViCs*, *traditional villages in Northern and Central Vietnam*, and *indigenous villages* remain analytically significant.

**Fig 7:**
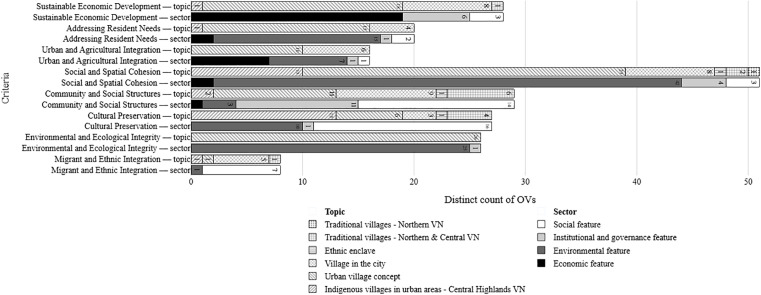
Number and distribution of OVs by criteria, literature topic and sector. Source: author.

In terms of distribution, the *Urban Village Concept* shows the broadest coverage, particularly dominating *Environment and Ecological Integrity* – the most emphasised criterion. *ViCs-*related variables appeared across the remaining seven criteria. *Indigenous villages* contribute strongly to *Social and Spatial Cohesion* and *Cultural Preservation*, while *traditional villages in Northern and Central Vietnam* enriched *Community and Social Structure*. (Fig 7)

### Assessing the hypothesised urban villages

Valid survey responses were obtained from 74 of 75 villages. One village (Da Thanh, Ward 7) yielded no valid responses due to logistical constraints arising from the large number of villages surveyed within a limited timeframe and was excluded from further analysis.

Using the context-based evaluation framework, 38 of 39 villages in Da Lat and 31 of 35 villages in the surrounding area scored above 50/100, with several exceeding 75 points. High-scoring villages include Da Loc, Da Trung, Da Thien, Ha Dong Flower Village, Hong Lac, Nam Ho, and Nam Thien in Da Lat, as well as Lac Thien, Lac Quang, Quang Lac, B’Neur 1, and B’Neur 2 in the vicinity. These results provide strong empirical support for the existence of *urban villages* in Da Lat and its vicinity.

Most villages exhibited substantial alignment with *ViCs*-related variables, while features derived from *traditional Kinh villages* and *indigenous villages* contributed differentially across criteria. Several settlements – including Mangline, Bac Hoi, Drong, and Kekaff – display hybrid characteristics. Although only three *ethnic enclave*-related OVs were retained, these variables were present in nearly all villages, confirming the widespread persistence of core enclave values.

*Urban Village Concept* variables were excluded from the assessment of existing villages. Consequently, all villages met only seven of the eight criteria, consistently lacking in *Environmental and Ecological Integrity*, which is defined exclusively by future-oriented planning variables. This highlights a critical ecological deficit in existing villages under current conditions.

**Fig 8:**
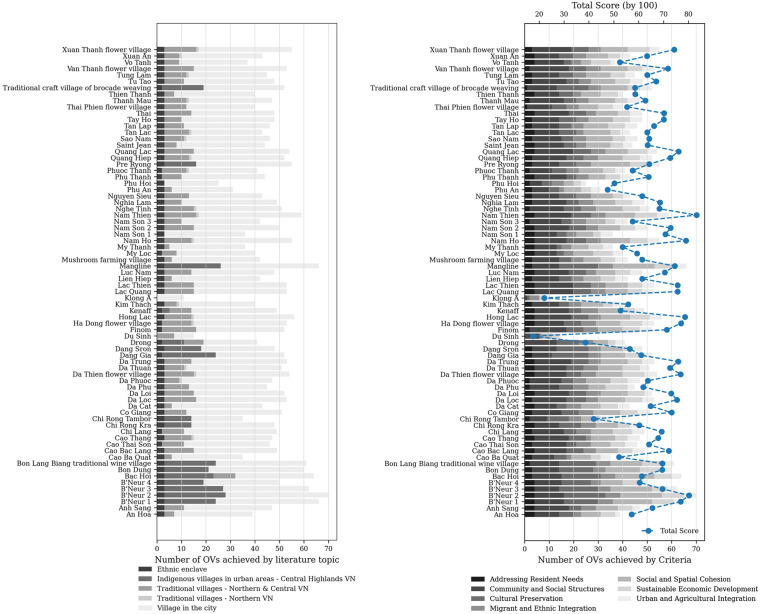
The observation of the hypothesised urban villages. Source: author.

Fig 8 visualises both the total scores (0–100) and the distribution of OVs across evaluation criteria for each *hypothesised urban village*. The bar charts illustrate the number of OVs met per criterion, while the overlaid line plot indicates the aggregated village score. Most villages display relatively balanced performances across multiple criteria, rather than excelling in a single dimension. High-scoring villages consistently combine social and spatial cohesion, community structures, and cultural preservation, despite limited ecological performance. Overall, Fig 8 highlights *urban villages* in Da Lat and its vicinity as socially embedded and contextually resilient formations, while also exposing their shared environmental limitations.

### Assessing the pilot project of Green Urban Village

When evaluated against the *Urban Village Concept* variables alone, the pilot *Green Urban Village* scored 65.78, indicating partial alignment with its reference model but also the absence of key elements such as public transport provision, prioritisation of walking and cycling, mixed-use integration, and coherent urban design guidelines.

When assessed against the full OVs set, the project scored 51.36. While it performed strongly in *Environmental and Ecological Integrity* – consistent with its green and high-tech agricultural vision – it showed weak performance in contextual dimensions, with only five variables related to traditional or indigenous villages identified.

**Fig 9:**
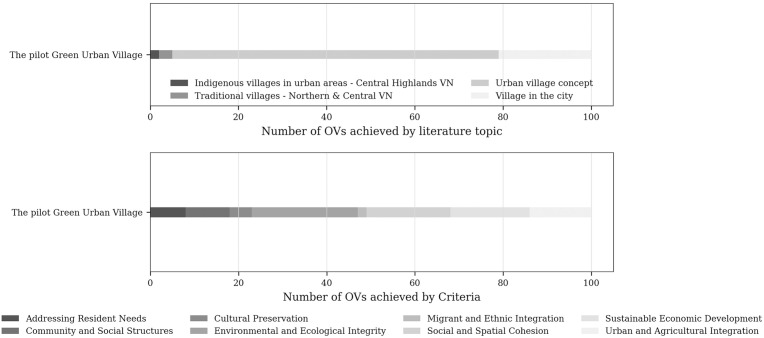
The observation of the pilot Green Urban Village. Source: author.

Fig 9 illustrates the criterion-level performance of the pilot project and reveals a markedly different profile from that of existing villages. In contrast to the more balanced distributions observed among *hypothesised urban villages*, the pilot project exhibits a strong concentration in environmental criteria and pronounced deficits in *Cultural Preservation*, *Community and Social Structures*, and *Migrant and Ethnic Integration*.

Across both evaluation frameworks, the pilot project does not constitute a viable *urban village model*. Its performance reveals limitations in both its translation of *urban village planning principles* and its integration with the socio-spatial context of Da Lat and its surrounding areas.

## Discussion

Taken together, Figs 8 and 9 demonstrate a clear contrast: while existing villages in Da Lat embody *urban village* characteristics through lived practices and historical continuity, the *Green Urban Village* pilot project remains largely decontextualised. This contrast prompts a re-examination of how the “urban village” is conceptualised, operationalised, and transferred across socio-spatial contexts.

### Coexistence of multiple urban village interpretations

Addressing the first research question, the findings confirm the coexistence of *ViCs* and the *Urban Village Concept* within Da Lat and its vicinity. Evidence for *ethnic enclaves* is numerically limited; however, the persistence of core attributes – such as strong social ties, adaptive capacity, and identity consciousness – suggests that *ethnic enclave* characteristics operate as foundational social dimensions rather than as a dominant or spatially discrete settlement form.

This has important theoretical implications. Rather than constituting parallel or competing typologies, the three interpretations function at different analytical levels: *ethnic enclaves* articulate social relations and identity, *ViCs* capture socio-spatial persistence under urbanisation, and the *Urban Village Concept* represents a normative planning aspiration. Their coexistence in Da Lat reflects a layered configuration in which social practices, spatial forms, and planning imaginaries intersect unevenly.

### Hybridity as a distinctive feature of Da Lat

Compared with cases commonly documented in the literature, Da Lat exhibits a distinctive form of hybridity. Unlike Global South contexts where *ViCs* are primarily defined by informality and redevelopment pressure, or Western settings where the *Urban Village Concept* is often realised through market-led regeneration, Da Lat’s villages reflect a prolonged co-evolution of colonial planning, internal migration, indigenous settlement, and state-led experimentation. The result is a set of adaptive socio-spatial systems embedded within an expanding urban fabric, challenging binary distinctions between “formal” and “informal” or “planned” and “organic” settlements.

### From conceptual integration to operationalisation: toward Green Urban Village 2.0

**Fig 10:**
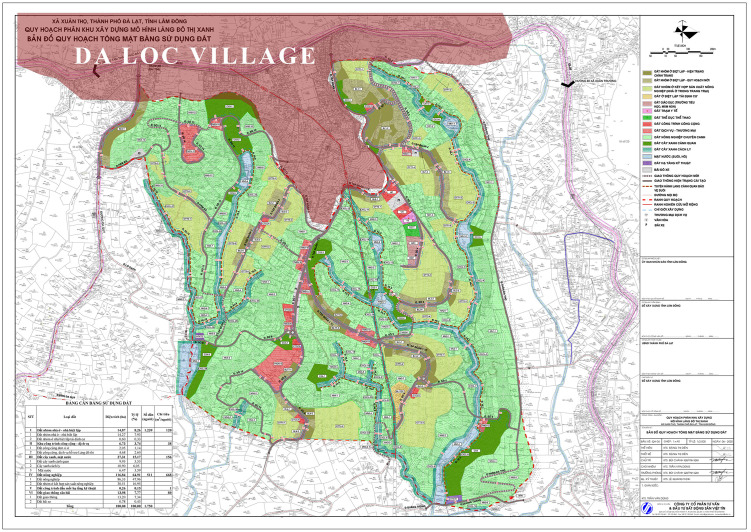
The boundary of the pilot Green Urban Village and the proximity of Da Loc village. Source: Adapted from [54] under a CC BY license, with permission from VITILAND, original copyright 2026.

**Fig 11:**
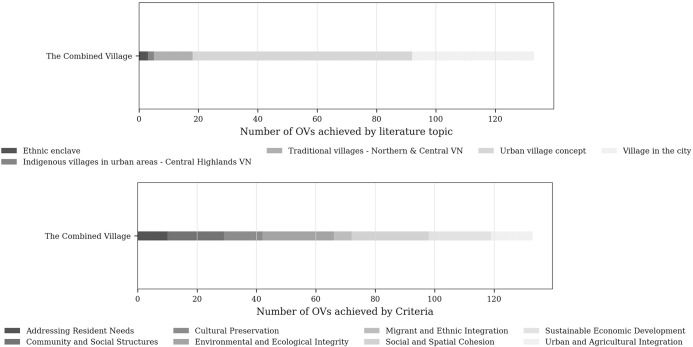
The indexes of the combined urban village. Source: author.

Addressing the second research question, the study shows that integration between *existing villages* and the *Green Urban Village* model is operationally plausible. The spatial proximity between the *pilot site* and *Da Loc village* provides a concrete illustration of such potential (Fig 10). A scenario-based combined assessment – analytically merging *Da Loc village* (score: 75.40) with the *pilot project* – yielded 133 OVs and an overall score of 67.92 distributed across all eight criteria (Fig 11). While not predictive, this exercise demonstrates how future-oriented planning interventions can be anchored in existing village structures. Compared with the pilot assessed in isolation, the integrated configuration displays a more balanced profile by compensating for socio-cultural deficits while reinforcing environmental dimensions.

Operationally, such integration does not require comprehensive redevelopment. In Da Loc, mixed-use integration could build on existing agricultural-residential interfaces through small-scale processing, local markets, and shared community facilities. Urban design interventions could prioritise pedestrian connectivity, incremental public space upgrades around communal institutions, and context-sensitive guidelines that respect existing parcel structures and everyday practices. In this sense, *Green Urban Village 2.0* is best understood not as a new settlement type, but as a relational model that enhances – rather than replaces – the socio-spatial logics of established villages.

### Methodological reflections, generalisability, and governance implications

The relatively high scores of existing villages may partly reflect measurement artefacts linked to binary scoring and the exclusion of future-oriented variables. While this limitation cannot be fully eliminated, it is mitigated through context-based variable selection, triangulation across multiple assessors, and consistent patterns across villages with diverse histories. The results should therefore be interpreted as indicative comparative patterns rather than definitive rankings.

Although grounded in Da Lat, the analytical framework may inform studies in other Global South contexts where informal persistence, internal migration, and experimental planning initiatives coexist, provided that criteria are recalibrated to local conditions.

Finally, the study carries implications for governance, particularly in light of Viet Nam’s administrative reforms enacted in July 2025, which introduce a two-tier local government structure. While these reforms do not alter the empirical findings, they may significantly affect the governance of *urban villages* by reshaping planning authority, coordination mechanisms, and community representation. Future research could explore how such institutional changes influence the capacity to implement hybrid models like *Green Urban Village 2.0*.

## Limitations

This study has several limitations.

First, villages were represented as point locations with a 500-m buffer rather than as precisely delineated spatial units. While adopted as a pragmatic solution to uncertainties in historical map georeferencing and boundary erosion under rapid urbanisation, this approach obscures fine-grained spatial variation. Future research could improve spatial precision by reconstructing historical village boundaries or incorporating parcel-level cadastral data.

Second, due to time constraints, thematic grouping of OVs using LDA was conducted after the first Delphi round, and the OVs list was finalised after the second round without a third round to reassess mid-range items. Although partially mitigated through expert weighting and EMO-optimised AHP, the absence of a final consensus round may have limited further convergence among expert judgments.

Third, field survey responses were recorded in binary form and aggregated using a majority rule. This enhanced inter-rater consistency and cross-site comparability but necessarily simplified complex socio-spatial phenomena and precluded sensitivity analysis. Accordingly, resulting scores should be interpreted as relative indicators rather than precise measurements.

Fourth, the scoring framework does not include confidence intervals or formal inferential statistical tests. Given the exploratory, case-based design and context-specific variable sets, robustness was sought through triangulation across multiple local assessors, transparent aggregation rules, and comparative interpretation rather than statistical generalisation. Future studies could strengthen robustness through probabilistic scoring, longitudinal data, or larger comparative samples.

Finally, the study was conducted prior to Vietnam’s administrative reforms officially enacted on July 1, 2025, which introduced a two-tier governance structure through the removal of the district level and consolidation of provinces. While the analytical framework and empirical findings remain valid, future research should examine how this institutional restructuring may affect planning authority, coordination, and participation in *urban village* development.

## Conclusion

This study demonstrates that the concept of the *urban village* – long contested within urban scholarship – can manifest in multiple, coexisting forms within a single territorial context. In Da Lat and its vicinity, the presence of *ViCs* and the *Urban Village Concept* was empirically confirmed, alongside the persistence of core attributes associated with *ethnic enclaves*. Rather than constituting mutually exclusive categories, these phenomena form a constellation of hybrid *urban village* forms shaped by historical migration, indigenous settlement practices, and contemporary planning imaginaries.

Methodologically, the study developed a context-sensitive evaluation framework to identify and assess 74 *hypothesised urban villages* across *eight criteria*. Most existing villages perform positively, underscoring their analytical relevance and practical significance in urban development. At the same time, the ecological dimension – largely defined through future-oriented planning variables – remains weakly developed, revealing a structural gap between lived village practices and aspirational sustainability agendas.

The assessment of the *Green Urban Village pilot project* highlights this gap. While the project aligns strongly with environmental objectives, it lacks contextual grounding and key planning elements such as mixed-use integration, public transport provision, pedestrian prioritisation, and coherent urban design standards. A scenario-based integrated assessment with the adjacent Da Loc village, however, yields a more balanced configuration across all eight criteria. This suggests that sustainability-oriented planning is more effective when anchored in existing village structures rather than implemented on vacant land. On this basis, the study proposes *Green Urban Village 2.0* – not as a new settlement typology, but as a relational and incremental model that enhances, rather than replaces, established socio-spatial logics.

Overall, the findings highlight the persistence and adaptability of *urban villages* under rapid urbanisation and call for planning frameworks that integrate local cultural practices, settlement histories, and social infrastructures. Future research should examine how such integrative approaches can be institutionalised and scaled within Vietnam’s evolving governance landscape. More broadly, this study contributes to debates on sustainable neighbourhoods and urban authenticity by showing how diverse interpretations of the *urban village* can function as complementary resources for context-sensitive urban futures.

## Supporting information

S1 Data OVs set after Round 1. (CSV)

S2 Data Finalised OVs. (CSV)

S3 Data Expert AHP matrices. (CSV)

S4 Data Scoring of villages in Da Lat. (CSV)

S5 Data Scoring of villages in the vicinity. (CSV)

S6 Data Scoring of the Green Urban Village. (CSV)

S1 File Code for criteria modeling using LDA. (ZIP)

S2 File Code for AHP and addressing inconsistencies using EMO. (ZIP)

S3 File Code for visualising number and distribution of OVs. (ZIP)

S4 File Code for villages assessments. (ZIP)

S1 Table Finalised observed variables. (DOCX)

S1 Text Note for supporting Codes. (DOCX)

S5 File Spatial data. (ZIP)
