# Supplementary material for: (Re)defining urban villages and their potential in sustaining local authenticity: A case study of Da Lat, Viet Nam
Source: PLoS One. 2026 Apr 3;21(4):e0345741. doi: 10.1371/journal.pone.0345741 (PMC13048443; doi:10.1371/journal.pone.0345741)
Supplement: S1 File — (ZIP) [file pone.0345741.s007.zip › S1_File.pdf]

1. INSTALL AND IMPORT LIBRARY

Library install

```
!pip install --upgrade --force-reinstall \
numpy<2.0 \
pandas==2.2.2 \
gensim==4.3.3 \
scipy \
scikit-learn \
nltk \
pyLDAvis
```

Show hidden output

Import library

```
import pandas as pd
import numpy as np
import nltk
import gensim
import gensim.corpora as corpora
import pyLDAvis
import pyLDAvis.gensim_models
import matplotlib.pyplot as plt
from nltk.corpus import stopwords
from nltk.stem import WordNetLemmatizer
from gensim.models.ldamodel import LdaModel
from sklearn.feature_extraction.text import CountVectorizer
import re

nltk.download('stopwords')
nltk.download('wordnet')
```

Show hidden output

2. IMPORT DATA

```
df=pd.read_csv('/content/ID_after_round_1_description_EN.csv')
df.head()
```

Show hidden output

3. TEXT PREPROCESSING

- Stopword removal
- Convert text to lowercase
- Tokenization

```
stop_words = set(stopwords.words('english')) #Defining stopword
stop_words.update(["many", "going", "still", "towards", "also", "etc", "must"]) #Update stopword
lemmatizer = WordNetLemmatizer() #Defining lemmatizer
```

Show hidden output

```
# (re)defining phrases to keep
phrases_to_keep = [
    "urban village",
    "mixed use",
    "public space",
    "informal settlement",
    "land use",
    "urban renewal",
    "community engagement",
    "housing policy"
]
```

```
    # Convert these phrases to the format "word1_word2"
    phrases_subs = {phrase: phrase.replace(" ", "_") for phrase in phrases_to_keep}

    def preprocess(text):
        # Replace these phrases
        for phrase, replacement in phrases_subs.items():
            text = re.sub(r'\b' + re.escape(phrase) + r'\b', replacement, text, flags=re.IGNORECASE)

        # Convert to lowercase and remove special characters
        text = re.sub(r'\W+', ' ', text.lower())

        # Split text
        tokens = text.split()

        # Lemmatize and remove stopwords
        tokens = [lemmatizer.lemmatize(word) for word in tokens if word not in stop_words and len(word) > 2]

        return tokens
```

Show hidden output

Additional explanation:

- `phrases_subs.items()` is a dictionary in the form: {"urban village": "urban_village", "mixed use": "mixed_use", ...}
  - The `.items()` function returns each pair (phrase, replacement) so we can iterate through each phrase.
- `for phrase, replacement in`: Iterates through each phrase that should be preserved as a single unit, for example:
  - phrase = "urban village"
  - replacement = "urban_village"
- The `re.sub()` function replaces strings that match a regular expression (regex) pattern within the text
  - `re.sub(pattern, replacement, text, flags)`
  - `pattern = r'\b' + re.escape(phrase) + r'\b'` creates a regular expression that ensures only the exact phrase is replaced, for example:
  - If phrase = "urban village", then: `re.escape(phrase)` → "urban\ village" (if special characters exist, they will be safely “escaped”).
  - \b at the beginning and end → r'\burban\ village\b'
  - \b is a word boundary: it ensures that "urban village" is matched only as a complete phrase, not as part of other words.
- `replacement`: For example "urban_village" → it will replace the matched "urban village" in the text.
- `flags=re.IGNORECASE` allows matching regardless of letter case (case-insensitive).

```
    df['tokens'] = df['Description_EN'].apply(preprocess)
```

Show hidden output

4. TRAINING LDA MODEL

Creating Dictionary and Corpus for LDA

Create the dictionary and corpus - the 2 input formats required by the LDA model in the Gensim library.

- `Dictionary`: maps words (tokens) to unique numerical IDs.
- `Corpus`: converts each document (a list of tokens) into a list of (word_id, word_count) pairs - i.e., a bag-of-words representation.

```
    id2word = corpora.Dictionary(df['tokens'])
    corpus = [id2word.doc2bow(text) for text in df['tokens']]
```

Show hidden output

Training model

```
    # Traing LDA model with X topics
    lda_model = LdaModel(
        corpus=corpus,
        id2word=id2word,
        num_topics=8,
```

```
        random_state=42,
        update_every=1,
        chunksize=100,
        passes=10,
        alpha='auto',
        per_word_topics=True
    )
```

Show hidden output

- This command instructs Gensim to train an LDA model with:
  - X topics
  - Preprocessed data (corpus + id2word)
  - 10 training passes, with a batch size of 100
  - Controlled randomness to ensure reproducibility
  - Outputting the topic distribution over words.

Optimize the number of topics using Coherence Score.

```
from gensim.models import CoherenceModel
import matplotlib.pyplot as plt

def compute_coherence_values(dictionary, corpus, texts, start=2, limit=20, step=1):
    coherence_values = []
    model_list = []

    for num_topics in range(start, limit + 1, step):
        model = LdaModel(
            corpus=corpus,
            id2word=dictionary,
            num_topics=num_topics,
            random_state=42,
            update_every=1,
            chunksize=100,
            passes=10,
            alpha='auto',
            per_word_topics=True
        )
        model_list.append(model)
        coherence_model = CoherenceModel(model=model, texts=texts, dictionary=dictionary, coherence='c_v')
        coherence_values.append(coherence_model.get_coherence())

    return model_list, coherence_values

# Run the function and plot the graph
model_list, coherence_values = compute_coherence_values(dictionary=id2word, corpus=corpus, texts=df['tokens'], start=2, limit=2

# Plot the graph
x = range(2, 21)
plt.plot(x, coherence_values)
plt.xlabel("Number of topics")
plt.ylabel("Coherence Score")
plt.title("Optimizing number of topics LDA")
plt.grid(True)
plt.show()
```

Show hidden output

5. RESULT

```
topics = lda_model.show_topics(num_topics=8, num_words=10, formatted=False)

for i, topic in topics:
    # Extracting words and weight
    words = [f"{word} ({weight:.4f})" for word, weight in topic]

    # Print topic with words and weights
    print(f"Topic {i}: {', '.join(words)}")
```

Show hidden output

```
pyLDAvis.enable_notebook()
vis = pyLDAvis.gensim_models.prepare(lda_model, corpus, id2word)
```

```
pyLDAvis.save_html(vis, 'lda_topic_visualization.html')
```

Show hidden output

```
topic_data = []

# Loop over each topic to extract words and weights
for i, topic in topics:
    words = [f"{word} ({weight:.4f})" for word, weight in topic]
    topic_str = ", ".join(words)

    # Assuming 'df' contains 'ID_new' and 'Description_EN'
    for index, row in df.iterrows():
        topic_data.append({
            'ID_new': row['ID_new'],
            'Description_EN': row['Description_EN'],
            'Topic_ID': i,
            'Topic_Description': topic_str
        })
```

Show hidden output

```
# Convert the data into a DataFrame
topic_df = pd.DataFrame(topic_data)

# Save to CSV file
#topic_df.to_csv("lda_topics_with_data.csv", index=False)
```

Show hidden output
