# Supplementary material for: (Re)defining urban villages and their potential in sustaining local authenticity: A case study of Da Lat, Viet Nam
Source: PLoS One. 2026 Apr 3;21(4):e0345741. doi: 10.1371/journal.pone.0345741 (PMC13048443; doi:10.1371/journal.pone.0345741)
Supplement: S2 File — (ZIP) [file pone.0345741.s008.zip › S2_File.pdf]

1. IMPORT LIBRARY & DATA

```
import pandas as pd
import numpy as np
```

```
url = "https://docs.google.com/spreadsheets/d/1xi4Ff5_Wrn37I7VsXiYj7wP7aoPUVokPyBeBcLiXs6A/export?format=csv"
#export?format=csv convert from google sheet to csv
df = pd.read_csv(url)
```

```
display(df.head())
display(df.tail())
```

Show hidden output

2. EDA & PREPROCESSING

```
df.info()
```

Show hidden output

3. CALCULATING AHP CONSISTENCE RATE

The parameters to be calculated:

- `Lambda_max`
- `CI (consistency index) = (Lamda_max - n)/(n-1)` with n is the number of criteria
- RI (from Saaty's table (1987))
- CR (consistency ratio) = CI/RI

3.1 Determine RI, taking parameters from Saaty's table (1987) with n = 8

```
# Number of criteria
n_criteria = 8

# RI dictionary according to Saaty
RI_dict = {1: 0.00, 2: 0.00, 3: 0.52, 4: 0.89, 5: 1.11, 6: 1.15, 7: 1.35, 8: 1.40} #according to Saaty (1987)

# RI (Random Index)
RI = RI_dict[n_criteria]
```

3.2. Calculating

Determine the formula

```
# Formula for calculating CR from an AHP matrix

def calculate_cr(matrix):
    matrix = np.array(matrix) # Convert the input into a NumPy array → helps perform mathematical operations more efficiently
    # Normalize the matrix by columns.
    # Each column of the matrix is divided by the sum of that column. Goal: make every column sum to 1
    normalized = matrix / matrix.sum(axis=0)
    # Priority vector: the average of each row in the normalized matrix
    priority_vector = normalized.mean(axis=1)
    # Compute the largest eigenvalue (λ_max) – using Saaty’s approach:
    ## Multiply the original matrix by the priority vector → then divide each element of the result by the corresponding element of the priority vector
    ## Take the average of these values
    lambda_max = (matrix @ priority_vector / priority_vector).mean()
    # CI
    CI = (lambda_max - n_criteria) / (n_criteria - 1)
    # CR
    CR = CI / RI
    return CR
```

Since the matrix is in fractional form such as 1/3, 1/5, 1/7, 1/9 => Convert the AHP value to a float (real number) form

```
def parse_value(x):
    x = str(x).strip().replace(",", ".") # Standardize the input x string
    if "/" in x:                         # Check whether the string is in fractional form
        num, denom = x.split("/")
        return float(num) / float(denom)
    return float(x)

# EXPLANATION:
## str(x) → ensures that x is a string (in case it is currently a number, NaN, etc.)
## strip() → removes extra whitespace at the beginning and end.
## replace(",", ".") → converts commas into decimal points
## num, denom = x.split("/"): If "/" is present, split the string into numerator and denominator. Example: "1/3" → num = "1", denom = "3"
## return float(num) / float(denom): Converts both numerator and denominator to floats and performs the division. Example: float("1") / float("3") = 0.333
```

Calculate the CR of each expert

- Calculate the CR of each expert (each expert is an 8-row x 8-column matrix)
- If CR < 0.10, the matrix is considered consistent.

```
# Initialize a list "results" to store the output
results = []

# Loop through the data in steps of n_criteria (8 rows at a time),
# because each AHP matrix is 8x8 and spans 8 consecutive rows for each expert
    ## Retrieve the "expert_name" from row i, first column
    ## Extract each 8x8 AHP matrix block "matrix_block":
    ## 8 rows from i to i+8 (since for i in range(0...) the first row of data (excluding the column header row) has index 0)
    ## 8 columns from C to J → corresponding to columns 2:10 because Python counts from 0
for i in range(0, len(df), n_criteria):
    expert_name = df.iloc[i, 0]
    matrix_block = df.iloc[i: i + n_criteria, 2: 2 + n_criteria]

# Compute CR
    ## map(parse_value): apply parsing based on string/fraction values already converted to floats
    ## values converts the result into a NumPy array for numerical computation
    try:
        matrix = matrix_block.map(parse_value).values
        cr = calculate_cr(matrix)
        results.append({"Expert": expert_name, "CR": round(cr, 4)}) # Store the result in the results[]
    ## Error handling (e.g., values cannot be converted to numbers): the expert is still recorded, but CR will be None
    except Exception as e:
        results.append({"Expert": expert_name, "CR": None, "Error": str(e)})

# Display results
df_results = pd.DataFrame(results) # Convert the results list into a DataFrame
print("🟢 CR results for each expert:")
print(df_results)

#Filter rows with CR > 0.10
num_adjust = df_results[df_results["CR"] > 0.10].shape[0] #shape[0] trả về số lượng dòng
print(f"\n🔴 Number of experts requiring adjustment (CR > 0.10): {num_adjust} / {len(df_results)}")

print("\n🔴 Experts requiring adjustment due to CR > 0.10:")
print(df_results[df_results["CR"] > 0.10])
```

Show hidden output

4. ADDRESSING INCONSISTENCY IN AHP - USING EMO

4.1. Install pymoo library to apply Multi-objective Optimization (NSGA-II)

```
!pip install pymoo==0.6.0
```

Show hidden output

```
import pymoo
```

```
from pymoo.core.problem import ElementwiseProblem   # Used to define a custom optimization problem
from pymoo.algorithms.moo.nsga2 import NSGA2        # NSGA-II optimization algorithm
from pymoo.optimize import minimize                 # Function to perform the optimization
from scipy.linalg import eig
from pymoo.operators.selection.rnd import RandomSelection
from pymoo.operators.crossover.sbx import SBX               # SBX crossover operator
from pymoo.operators.mutation.pm import PolynomialMutation  # Polynomial mutation operator
```

4.2. Building computational functions

Build the function `rebuild_matrix`: reconstruct the matrix from the optimized values.

- Purpose: This function is used to rebuild the full AHP matrix (symmetric with 1 on the diagonal) from a vector containing the elements above the main diagonal.

```
# The function receives:
## x: a list (or array) containing the values above the main diagonal of the AHP matrix,
##    generated by the optimization process (with length n(n-1)/2).
## n: the number of criteria → the matrix size will be n x n.
def rebuild_matrix(x, n):

    ## Create an n x n matrix with all elements = 1.
    ## The main diagonal in an AHP matrix is always 1, so this is a reasonable initialization
    matrix = np.ones((n, n))

    ## Initialize the counter variable idx to sequentially retrieve values from vector x
    idx = 0

    ## Loop:
        ### Iterate through indices in the upper triangular part (only where i < j)
        ### Total iterations = n(n-1)/2, exactly the number of elements in x
    for i in range(n):
        for j in range(i+1, n):
```

```
            #### Assign the value from vector x to position [i][j] of the matrix (above the diagonal)
            matrix[i][j] = x[idx]
            #### AHP requires the matrix to follow the reciprocal symmetry rule
            #### → The opposite position [j][i] is the reciprocal of [i][j]
            matrix[j][i] = 1 / x[idx]
            #### Increase idx to retrieve the next value from vector x
            idx += 1
    return matrix
```

Define a multi-objective optimization problem for the NSGA-II algorithm to adjust the AHP matrix. The overall objectives are:

- Optimize the vector x (containing the values of the upper triangular part of the AHP matrix) to produce a valid AHP matrix.
- Reduce the CR (Consistency Ratio).
- Keep the matrix as close as possible to the original matrix (measured using the Euclidean norm of the deviation).

```
# Initialize the class
class AHPRepairProblem(ElementwiseProblem): # The class inherits from pymoo's ElementwiseProblem, optimizing each individual separately (element-wise)
    def __init__(self, original_matrix):    # Receives an initial AHP matrix (original_matrix) from a given expert
        self.n = original_matrix.shape[0]   # Store the number of criteria (n) of the original matrix
        self.original_matrix = original_matrix  # Store a copy of the original matrix

        ## n_var   Number of variables to optimize = number of elements in the upper triangular part of the AHP matrix (n(n-1)/2)
        ## n_obj=2 There are 2 objective functions: (1) minimize CR and (2) minimize deviation from the original matrix
        ## xl=1/9  Lower bound (minimum value) for each variable (AHP scale rule: from 1/9 to 9)
        ## xu=9    Upper bound
        super().__init__(n_var=int(self.n * (self.n - 1) / 2),
                         n_obj=2,
                         xl=1 / 9,
                         xu=9)

    def _evaluate(self, x, out, *args, **kwargs): # This function is used whenever the algorithm needs to evaluate the "fitness" of a candidate vector x
        matrix = rebuild_matrix(x, self.n)        # Reconstruct the full AHP matrix from vector x (values above the diagonal)
        cr = calculate_cr(matrix)                 # Compute the CR of the new matrix

        # Compute the difference between the optimized matrix and the original matrix
        # (using the Euclidean norm – the square root of the sum of squared differences)
        diff = np.linalg.norm(matrix - self.original_matrix)
        out["F"] = [cr, diff]
```

4.3. Optimize AHP matrix

```
'''
Function optimize_all_ahp_matrices:
inputs:
- df: dataframe containing the entire original AHP dataset (including the 8x8 matrix blocks of each expert).
- df_results: the initial CR calculation results for each expert (generated in the previous section).
- output_path: the file path for saving the optimization results as a CSV file.
'''

def optimize_all_ahp_matrices(df, df_results, output_path="optimized_results.csv"):

    # Step 1: Initialize an empty results list
    results_MOO = []
    # Step 2: Loop through each expert
    for _, row in df_results.iterrows():
        expert = row["Expert"]
        original_cr = row["CR"]
    # Step 3: Extract the expert's original matrix
        ## Filter the expert's 8x8 AHP matrix block.
        ## Use parse_value to convert from string/fraction format to floating-point numbers
        expert_data = df[df["Expert"] == expert]
        matrix = expert_data.iloc[:, 2:2 + n_criteria].applymap(parse_value).to_numpy()

        # Check the expert's initial CR
        ## If CR is NaN or excessively high, skip the optimization.
        ## Record the information in results_MOO and continue to the next expert
        if pd.isna(original_cr) or original_cr > 1:
            results_MOO.append({
                "Expert": expert,
                "Original_CR": original_cr,
                "Optimized_CR": None,
                "Delta_CR": None,
                "Error": "Invalid data or excessively high CR"
            })
            continue

        # Initialize the optimization problem
        ## Use the AHPRepairProblem class to define the optimization objectives:
        ## reduce CR while preserving the original structure as much as possible
        problem = AHPRepairProblem(matrix)

        # Configure the NSGA-II algorithm
        algorithm = NSGA2(
            pop_size=50,                                   # population size: number of individuals in each generation
            selection = RandomSelection(),                 # randomly select pairs of individuals to generate the next generation
            crossover = SBX(),                             # crossover operator (Simulated Binary Crossover)
            mutation = PolynomialMutation(prob=1.0, eta=20),  # mutation: randomly modify some variables in an individual
            eliminate_duplicates=True                      # remove duplicate solutions
        )

        # Run the optimization
        res = minimize(problem,
                       algorithm,
                       ('n_gen', 100),  # Optimize for 100 generations to find a solution with the lowest CR
```

```
                       seed=1,
                       verbose=False)

        # Find the best solution (lowest CR)
        best_idx = np.argmin([calculate_cr(rebuild_matrix(x.X, n_criteria)) for x in res.pop])
        best_solution = res.pop[best_idx].X

        # Rebuild the matrix and compute the CR after optimization
        best_matrix = rebuild_matrix(best_solution, n_criteria)
        optimized_cr = calculate_cr(best_matrix)

        # Flatten and store the optimized matrix results
        flattened_matrix = best_matrix.flatten()
        matrix_dict = {f"m_{i}_{j}": best_matrix[i, j] for i in range(n_criteria) for j in range(n_criteria)}

        results_MOO.append({
            "Expert": expert,
            "Original_CR": original_cr,
            "Optimized_CR": optimized_cr,
            "Delta_CR": original_cr - optimized_cr,
            **matrix_dict
        })

    # Save the results to a CSV file
    results_MOO_df = pd.DataFrame(results_MOO)
    results_MOO_df.to_csv(output_path, index=False)
    print("✅ Results saved to:", output_path)
    return results_MOO_df
# Call the function to process and optimize all AHP matrices
results_MOO_df = optimize_all_ahp_matrices(df, df_results)
print("🟢 CR results for each expert:")
print(results_MOO_df)
```

Show hidden output

NOTES:

- Population size in the AHP context: Each individual is a vector representing a repaired AHP matrix (with n(n−1)/2 variables corresponding to the values in the upper triangular part of the matrix).
- `selection = RandomSelection()`: In multi-objective problems such as AHP, diversity can be prioritized rather than always selecting only the best solutions.
- Crossover operator: Creates offspring by combining two parent individuals.
    - SBX (Simulated Binary Crossover) simulates binary crossover behavior in a continuous search space (since the variables here are real numbers).
    - Purpose: Explore the solution space by exchanging information between candidate solutions.
- `mutation = PolynomialMutation(prob=1.0, eta=20)`:
    - prob=1.0: Mutation is applied to every individual.
    - eta=20: A parameter controlling the spread of the mutation:
    - Smaller eta ⟶ stronger mutation (far from the original value).
    - Larger eta ⟶ weaker mutation (closer to the original value).
    - Purpose: Helps avoid getting trapped in local optima and increases solution diversity.
- `eliminate_duplicates=True`:
    - Meaning: Identical individuals are not allowed to exist in the population.
    - Purpose: Prevents duplicate solutions and keeps the evolutionary process efficient and diverse.

5. CALCULATING TOPIC WEIGHT

```
# Filter matrix column from results_df
matrix_column = [f"m_{i}_{j}" for i in range(n_criteria) for j in range(n_criteria)]
matrices = results_MOO_df[matrix_column].to_numpy().reshape(-1, n_criteria, n_criteria)
```

```
from scipy.stats import gmean
```

```
# Compute the geometric mean for each pair (i, j)
aggregated_matrix = gmean(matrices, axis=0)
```

```
aggregated_cr = calculate_cr(aggregated_matrix)
print(f" CR of aggregated matrix from 25 experts: {aggregated_cr:.4f}")
```

```
 CR of matrix from 25 experts: 0.0002
```

```
criteria_names = df.columns[2:2 + n_criteria].tolist()

# Normalize the aggregated matrix
normalized = aggregated_matrix / aggregated_matrix.sum(axis=0)

# Compute the weights as the row-wise average
priority_vector = normalized.mean(axis=1)
```

```
# Create the weights table
weights_df = pd.DataFrame({
    "Criterion": criteria_names,
    "Weight": priority_vector
})
```

```
# Add a column for weight percentage
weights_df["Weight (%)"] = (weights_df["Weight"] * 100).round(2)  # round to 2 decimal places

# Sort in descending order by weight
weights_df = weights_df.sort_values(by="Weight", ascending=False).reset_index(drop=True)

print("Weights and priority ranking of the criteria:")
print(weights_df)
```

Show hidden output
