# Supplementary material for: (Re)defining urban villages and their potential in sustaining local authenticity: A case study of Da Lat, Viet Nam
Source: PLoS One. 2026 Apr 3;21(4):e0345741. doi: 10.1371/journal.pone.0345741 (PMC13048443; doi:10.1371/journal.pone.0345741)
Supplement: S3 File — (ZIP) [file pone.0345741.s009.zip › S3_File.pdf]

IMPORT LIBRARY AND DATA

```
import pandas as pd
import numpy as np
```

```
url = "https://docs.google.com/spreadsheets/d/14MrzpCvJFQynvnL50w-SIoCJWsi5i-idCUoxifGD2n4/export?format=csv"
df = pd.read_csv(url)
```

```
display(df.head(3))
display(df.tail(3))
```

Show hidden output

```
df.info()
```

```
<class 'pandas.core.frame.DataFrame'>
RangeIndex: 205 entries, 0 to 204
Data columns (total 9 columns):
 #   Column                  Non-Null Count  Dtype
---  ------                  --------------  -----
 0   ID_new                  205 non-null    object
 1   Category_EN             205 non-null    object
 2   Sub_category_EN         205 non-null    object
 3   Final_decision          205 non-null    object
 4   Average mean            205 non-null    object
 5   Average mean format     205 non-null    float64
 6   Description             205 non-null    object
 7   Description_EN_new      205 non-null    object
 8   Criteria designation_EN  205 non-null    object
dtypes: float64(1), object(8)
memory usage: 14.5+ KB
```

STACKED BAR CHART

```
# Make sure ID_new is string, before calculating nunique
df['ID_new'] = df['ID_new'].astype(str)
```

```
# count distinct ID_new according to (Criteria, Category) and according to (Criteria, Sub_category)
cat_id_sum = (
    df.groupby(['Criteria designation_EN', 'Category_EN'])['ID_new']
      .nunique()
      .reset_index()
      .rename(columns={'ID_new': 'Distinct_ID_count'})
)

sub_id_sum = (
    df.groupby(['Criteria designation_EN', 'Sub_category_EN'])['ID_new']
      .nunique()
      .reset_index()
      .rename(columns={'ID_new': 'Distinct_ID_count'})
)
```

```
# Use the name of Category/Subcategory as label for legend
cat_id_sum['type'] = cat_id_sum['Category_EN']
sub_id_sum['type'] = sub_id_sum['Sub_category_EN']

# y label
cat_id_sum['y'] = cat_id_sum['Criteria designation_EN'] + " – topic"
sub_id_sum['y'] = sub_id_sum['Criteria designation_EN'] + " – sector"

# Combine them and rename the count column to 'x'.
plot_df = pd.concat([
    cat_id_sum[['y', 'Distinct_ID_count', 'type']],
    sub_id_sum[['y', 'Distinct_ID_count', 'type']]
], ignore_index=True).rename(columns={'Distinct_ID_count': 'x'})

# Arrange the rows in order so that each Criteria has two rows: Category (top) then Sub_category (bottom).
criteria_order = df['Criteria designation_EN'].drop_duplicates().tolist()
y_order = []
for c in criteria_order:
    y_order.append(f"{c} – topic")
    y_order.append(f"{c} – sector")
```

```
import plotly.express as px
import plotly.graph_objects as go
import plotly.io as pio
pio.renderers.default = 'colab'   # Ensure display in Colab
```

```
# List of grayscale colors (will be iterated over if the number of categories > the number of colors)
gray_colors = ['#000000', '#666666', '#CCCCCC', '#FFFFFF']

# List of pattern shapes (will loop if necessary)
pattern_shapes = ['/', '\\', 'x', '-', '+', '.', '|']

# Retrieve legend values for each group (based on existing cat_id_sum & sub_id_sum)
topic_types = list(cat_id_sum['type'].astype(str).unique())
sector_types = list(sub_id_sum['type'].astype(str).unique())
```

```
fig = go.Figure()
```

```
# Literature topic (patterned fill)
for i, t in enumerate(topic_types):
    df_t = plot_df[(plot_df['type'] == t) & (plot_df['y'].str.endswith('topic'))]
    if df_t.empty:
        continue
    fig.add_trace(go.Bar(
        x = df_t['x'],
        y = df_t['y'],
        name = str(t),
        legendgroup="topic",
        orientation = 'h',
        marker = dict(
            color = 'white',  # background fill (pattern will appear on this background)
            line = dict(color='black', width=0.8),
            pattern = dict(
                shape = pattern_shapes[i % len(pattern_shapes)],
                fgcolor = 'black',
                size = 4,
                solidity = 0.3
            )
        ),
        text = df_t['x'],
        hovertemplate = 'Criteria: %{y}<br>Category: ' + str(t) + '<br>Distinct IDs: %{x}<extra></extra>'
    ))

# Sector (grayscale, solid fill)
for i, t in enumerate(sector_types):
    df_t = plot_df[(plot_df['type'] == t) & (plot_df['y'].str.endswith('sector'))]
    if df_t.empty:
        continue
    fig.add_trace(go.Bar(
        x = df_t['x'],
        y = df_t['y'],
        name = str(t),
        legendgroup="sector",   # assign to sector group
        orientation = 'h',
        marker = dict(
            color = gray_colors[i % len(gray_colors)],
            line = dict(color='black', width=0.8)
        ),
        text = df_t['x'],
        hovertemplate = 'Criteria: %{y}<br>Sector: ' + str(t) + '<br>Distinct IDs: %{x}<extra></extra>'
    ))
```

```
# Layout & styling: Times New Roman, monochrome look, reverse the order of y according to y_order
fig.update_layout(
    barmode = 'stack',   # Stack values within the same y (Category/Subcategory values will stack according to their corresponding y)
    yaxis = dict(categoryorder='array', categoryarray=list(reversed(y_order)), automargin=True),
    xaxis_title = 'Distinct count of OVs',
    yaxis_title = 'Criteria',
    height = max(600, len(y_order) * 28),  # Dynamic height based on number of rows (minimum 600 px)
    font = dict(family='Times New Roman', size=14, color='black'),
    plot_bgcolor = 'white',
    paper_bgcolor = 'white',
    legend_title = '',
)

# Dummy trace for group headers
fig.add_trace(go.Bar(
    x=[None], y=[None],
    name="<b>Topic</b>",  # group's topic
    legendgroup="topic",
    marker=dict(color='rgba(0,0,0,0)'),
    showlegend=True
))

fig.add_trace(go.Bar(
    x=[None], y=[None],
    name="<b>Sector</b>",  # group's topic
    legendgroup="sector",
    marker=dict(color='rgba(0,0,0,0)'),
    showlegend=True
))

# Layout legend
fig.update_layout(
    legend=dict(
        orientation="h",
        yanchor="top",
        y=-0.15,
        xanchor="center",
        x=0.5,
        tracegroupgap=30  # distance between 2 groups
    )
)

# Text style for number on bar
fig.update_traces(textposition='auto', textfont=dict(family='Times New Roman', size=11, color='black'))

# Tweak axes lines / grid (light to be suitable to journal)
fig.update_xaxes(showgrid=True, gridcolor='lightgrey', zeroline=True, zerolinecolor='lightgrey')
fig.update_yaxes(showgrid=False)

fig.show()
```

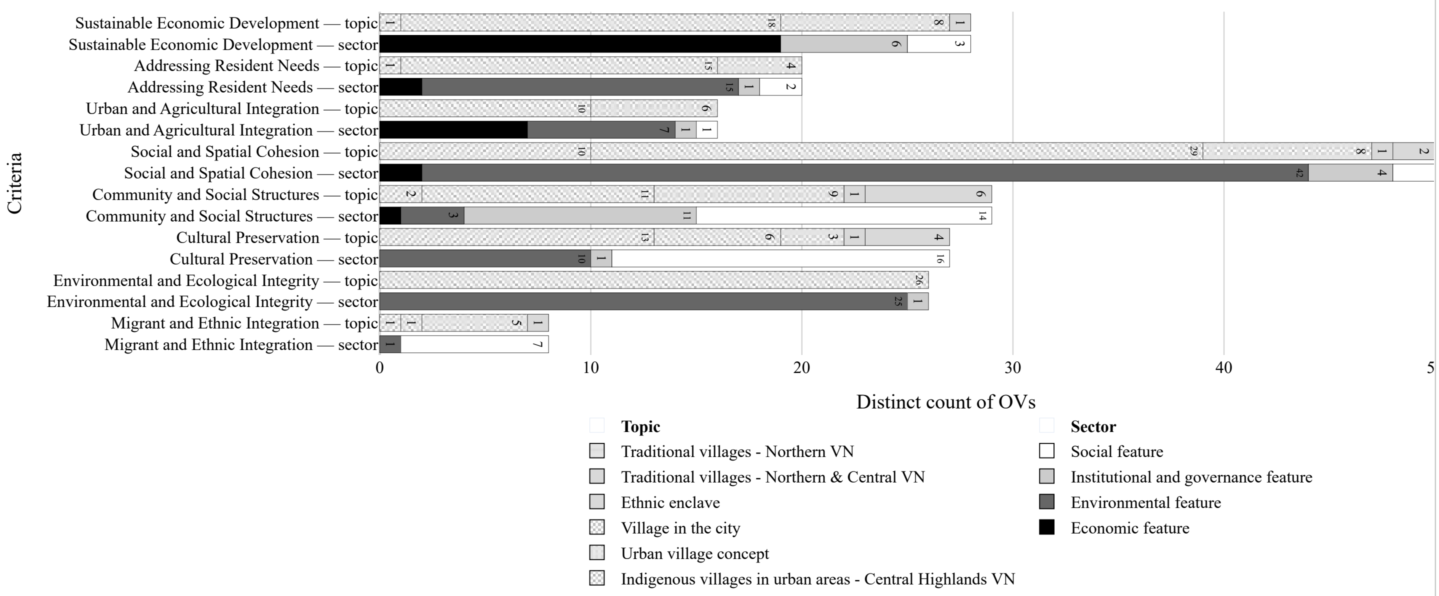
Criteria
Sustainable Economic Development — topic
Sustainable Economic Development — sector
Addressing Resident Needs — topic
Addressing Resident Needs — sector
Urban and Agricultural Integration — topic
Urban and Agricultural Integration — sector
Social and Spatial Cohesion — topic
Social and Spatial Cohesion — sector
Community and Social Structures — topic
Community and Social Structures — sector
Cultural Preservation — topic
Cultural Preservation — sector
Environmental and Ecological Integrity — topic
Environmental and Ecological Integrity — sector
Migrant and Ethnic Integration — topic
Migrant and Ethnic Integration — sector
Distinct count of OVs
Topic
Traditional villages - Northern VN
Traditional villages - Northern & Central VN
Ethnic enclave
Village in the city
Urban village concept
Indigenous villages in urban areas - Central Highlands VN
Sector
Social feature
Institutional and governance feature
Environmental feature
Economic feature
