# Supplementary material for: (Re)defining urban villages and their potential in sustaining local authenticity: A case study of Da Lat, Viet Nam
Source: PLoS One. 2026 Apr 3;21(4):e0345741. doi: 10.1371/journal.pone.0345741 (PMC13048443; doi:10.1371/journal.pone.0345741)
Supplement: S4 File — (ZIP) [file pone.0345741.s010.zip › S4_File.pdf]

1. Import Data

```
import pandas as pd
import numpy as np
```

```
url_OVs = "https://docs.google.com/spreadsheets/d/14MrzpCvJFQynvnL50w-SIoCJWsi5i-idCUoxifGD2n4/export?format=csv"
url_dalat = "https://docs.google.com/spreadsheets/d/1TnJgwqN5oV-d55RF13GNeH9m26gUZg5vbhoV-dNdtFY/export?format=csv"
url_vicinity = "https://docs.google.com/spreadsheets/d/1zLVOPzvEQ3YFgicTKHKHBoF2RRUMDT_36TuR7Kvhpvg/export?format=csv"
url_GUV = "https://docs.google.com/spreadsheets/d/1ZWilS5KMrHjgyvf7GiBV-nTebiEy-NFnBmGGHavZxTg/export?format=csv"
```

```
df_OVs = pd.read_csv(url_OVs)
df_dalat = pd.read_csv(url_dalat)
df_vicinity = pd.read_csv(url_vicinity)
df_GUV = pd.read_csv(url_GUV)
```

2. EDA & Proprocessing

```
display(df_OVs.info())
display(df_dalat.info())
display(df_vicinity.info())
display(df_GUV.info())
```

Show hidden output

```
display(df_OVs.head(3))
display(df_dalat.head(3))
display(df_vicinity.head(3))
display(df_GUV.head(3))
```

Show hidden output

```
#Assign float64 columns to int64 (int will throw an error if there is NaN, so it must be Int64)
cols = ['Phiếu 1', 'Phiếu 2',   'Phiếu 3',  'Phiếu 4',  'Phiếu 5',  'Phiếu 6',  'Phiếu 7']
df_dalat[cols] = df_dalat[cols].astype("Int64")
df_vicinity[cols] = df_vicinity[cols].astype("Int64")
```

```
display(df_dalat.head(3))
display(df_vicinity.head(3))
```

Show hidden output

3. Filter by context

3.1. Dalat & Vicinity

```
# Voting columns
vote_cols = ["Phiếu 1", "Phiếu 2", "Phiếu 3", "Phiếu 4", "Phiếu 5", "Phiếu 6", "Phiếu 7"]
```

```
# Calculate the average and generate the survey result
## Đà Lạt
df_dalat["average"] = df_dalat[vote_cols].mean(axis=1, skipna=True).round(2)
## Vicinity
df_vicinity["average"] = df_vicinity[vote_cols].mean(axis=1, skipna=True).round(2)

# Defining Survey result
def survey_result(val):
    if pd.isna(val):
        return "invalid"
    elif val > 0.5:
        return "true"
    elif val < 0.5:
        return "false"
    else:
        return "invalid"

## Dalat Survey result
df_dalat["Survey result"] = df_dalat["average"].apply(survey_result)
## Vicinity Survey result
df_vicinity["Survey result"] = df_vicinity["average"].apply(survey_result)
```

```
# Đà Lạt meeting OVs
# Step 1: filter data from df_dalat
filtered_dalat = df_dalat[
    (df_dalat["Survey result"] == "true") &
    (df_dalat["ID_new"].isin(df_OVs["ID_new"]))
]

# Step 2: merge to take Category, Sub_category, Criteria Designation from df_OVs
village_DaLat_meeting_variable = filtered_dalat.merge(
    df_OVs[["ID_new", "Category_EN", "Sub_category_EN", "Criteria designation_EN"]],
    on="ID_new",
    how="left"
```

```
)

# Step 3: choosing necessary columns only
village_DaLat_meeting_variable = village_DaLat_meeting_variable[
    ["Village", "ID_new", "Category_EN", "Sub_category_EN", "Criteria designation_EN"]
]
```

```
# Vicinity meeting OVs
# Step 1: filter data from df_vicinity
filtered_vicinity = df_vicinity[
    (df_vicinity["Survey result"] == "true") &
    (df_vicinity["ID_new"].isin(df_OVs["ID_new"]))
]

# Step 2: merge to take Category, Sub_category, Criteria Designation from df_OVs
village_vicinity_meeting_variable = filtered_vicinity.merge(
    df_OVs[["ID_new", "Category_EN", "Sub_category_EN", "Criteria designation_EN"]],
    on="ID_new",
    how="left"
)

# Step 3: choosing necessary columns only
village_vicinity_meeting_variable = village_vicinity_meeting_variable[
    ["Village", "ID_new", "Category_EN", "Sub_category_EN", "Criteria designation_EN"]
]
```

```
display(village_DaLat_meeting_variable.head(3))
display(village_vicinity_meeting_variable.head(3))
```

Show hidden output

3.2. Filter GUV

```
# Dont need to calculate average (only 1 scoring column by the author)
# Reuse the Survey result definition already provided above
''' def survey_result(val):
    if pd.isna(val):
        return "invalid"
    elif val > 0.5:
        return "true"
    elif val < 0.5:
        return "false"
    else:
        return "invalid" '''
# Survey result GUV
df_GUV["Survey result"] = df_GUV["Observation"].apply(survey_result)
```

3.2.1. Filter by total list

```
# GUV meeting OVs total list
# Step 1: filter data from df_GUV
filtered_GUV_total = df_GUV[
    (df_GUV["Survey result"] == "true") &
    (df_GUV["ID_new"].isin(df_OVs["ID_new"]))
]

# Step 2: merge to take Category, Sub_category, Criteria Designation from df_OVs
village_GUV_meeting_total = filtered_GUV_total.merge(
    df_OVs[["ID_new", "Category_EN", "Sub_category_EN", "Criteria designation_EN"]],
    on="ID_new",
    how="left"
)

# Step 3: choosing necessary columns only
village_GUV_meeting_total = village_GUV_meeting_total[
    ["Village", "ID_new", "Category_EN", "Sub_category_EN", "Criteria designation_EN"]
]
```

```
display(village_GUV_meeting_total.head(3))
display(village_GUV_meeting_total.tail(3))
```

Show hidden output

3.2.2. Filter by Urban Village Concept

```
# GUV meeting Urban Village Concept from OVs list
# Step 1: filter data from df_GUV
filtered_GUV_UVC = df_GUV[
    (df_GUV["Survey result"] == "true") &
    (df_GUV["ID_new"].isin(
        df_OVs.loc[df_OVs["Category_EN"] == "Urban village concept", "ID_new"]
    ))
]
# Step 2: merge to take Category, Sub_category, Criteria Designation from df_OVs
village_GUV_meeting_UVC = filtered_GUV_UVC.merge(
    df_OVs[["ID_new", "Category_EN", "Sub_category_EN", "Criteria designation_EN"]],
    on="ID_new",
    how="left"
)
```

```
# Step 3: choosing necessary columns only
village_GUV_meeting_UVC = village_GUV_meeting_UVC[
    ["Village", "ID_new", "Category_EN", "Sub_category_EN", "Criteria designation_EN"]
]
```

```
display(village_GUV_meeting_UVC.head(3))
display(village_GUV_meeting_UVC.tail(3))
```

Show hidden output

4. Develop a scoring formula

4.1. Create criteria_weight dataframe

```
# Create criteria_weight dataframe
criteria_weight = pd.DataFrame({
    "Criterion_EN": [
        "Cultural Preservation",
        "Urban and Agricultural Integration",
        "Environmental and Ecological Integrity",
        "Sustainable Economic Development",
        "Social and Spatial Cohesion",
        "Addressing Resident Needs",
        "Community and Social Structures",
        "Migrant and Ethnic Integration"
    ],
    "Weight_percentage": [15.62, 14.12, 13.65, 12.44, 12.32, 12.21, 11.93, 7.71]
})
```

```
print(criteria_weight)
```

Show hidden output

4.2. Scoring formula

```
# Define a scoring function for each Village
def compute_scores(village_name, criteria_weight, village_df, survey_df):
    results = []

    # subset of data for the current village
    v_df = village_df[village_df["Village"] == village_name]

    # categories that this village participates in
    valid_categories = v_df["Category_EN"].unique()

    for _, row in criteria_weight.iterrows():
        criterion = row["Criterion_EN"]
        weight = row["Weight_percentage"]

        # Number of ID_new matched
        num_matched = v_df.loc[v_df["Criteria designation_EN"] == criterion, "ID_new"].nunique()

        # The total number of ID_new for this criterion, but only within valid categories
        total_variables = survey_df.loc[
            (survey_df["Criteria designation_EN"] == criterion) &
            (survey_df["Category_EN"].isin(valid_categories)),
            "ID_new"
        ].nunique()

        if total_variables > 0 and num_matched > 0:
            score = (num_matched / total_variables) * weight
        else:
            score = None  # corresponds to BLANK() in Power BI

        results.append({
            "Village": village_name,
            "Criterion_EN": criterion,
            "Score": score
        })

    return pd.DataFrame(results)
```

4.3. Scoring

```
# Đà Lạt:
Dalat_scores = pd.concat([
    compute_scores(v, criteria_weight, village_DaLat_meeting_variable, df_OVs)
    for v in village_DaLat_meeting_variable["Village"].unique()
])
```

```
# Vicinity
Vicinity_scores = pd.concat([
    compute_scores(v, criteria_weight, village_vicinity_meeting_variable, df_OVs)
    for v in village_vicinity_meeting_variable["Village"].unique()
])
```

```
display(Dalat_scores.head(8))
```

```
display(Dalat_scores.tail(8))
```

Show hidden output

```
Dalat_scores['Village'].nunique()
```

```
39
```

```
display(Vicinity_scores.head(8))
display(Vicinity_scores.tail(8))
```

Show hidden output

```
Vicinity_scores['Village'].nunique()
```

```
35
```

```
# GUV by total:
GUV_total_scores = pd.concat([
    compute_scores(v, criteria_weight, village_GUV_meeting_total, df_OVs)
    for v in village_GUV_meeting_total["Village"].unique()
])
```

```
# GUV by UVC:
GUV_UVC_scores = pd.concat([
    compute_scores(v, criteria_weight, village_GUV_meeting_UVC, df_OVs)
    for v in village_GUV_meeting_UVC["Village"].unique()
])
```

```
display(GUV_total_scores.head(10))
display(GUV_UVC_scores.head(10))
```

Show hidden output

5. Visualize

5.1. Construct the function

5.1.1. Group Da Lat and vicinity - by literature topic (to count the number of OVs meeting)

```
# Đà Lạt
dalat_grouped_Category_EN = (
    village_DaLat_meeting_variable
    .groupby(["Village", "Category_EN"])
    .size()
    .reset_index(name="Count")
)
dalat_grouped_Category_EN["Source"] = "Da Lat"
# Vicinity
vicinity_grouped_Category_EN = (
    village_vicinity_meeting_variable
    .groupby(["Village", "Category_EN"])
    .size()
    .reset_index(name="Count")
)
vicinity_grouped_Category_EN["Source"] = "Vicinity"

# Combine Đà Lạt & Vicinity
combined_Category_EN = pd.concat([dalat_grouped_Category_EN, vicinity_grouped_Category_EN], ignore_index=True)
```

5.1.2. Group Da Lat and vicinity - by Criteria (to count the number of OVs meeting)

```
# Đà Lạt
dalat_grouped_Criteria_designation_EN = (
    village_DaLat_meeting_variable
    .groupby(["Village", "Criteria designation_EN"])
    .size()
    .reset_index(name="Count")
)
dalat_grouped_Criteria_designation_EN["Source"] = "Da Lat"
# Vicinity
vicinity_grouped_Criteria_designation_EN = (
    village_vicinity_meeting_variable
    .groupby(["Village", "Criteria designation_EN"])
    .size()
    .reset_index(name="Count")
)
vicinity_grouped_Criteria_designation_EN["Source"] = "Vicinity"

# Combine Đà Lạt & Vicinity
combined_Criteria_designation_EN = pd.concat(
    [dalat_grouped_Criteria_designation_EN, vicinity_grouped_Criteria_designation_EN], ignore_index=True
)
```

5.1.3. Create a dataframe for the total scores of villages in Da Lat and the vicinity

```
# Label the source (For classification when necessary)
Dalat_scores["Source"] = "Da Lat"
Vicinity_scores["Source"] = "Vicinity"

# Combine
combine_score_Dalat_vicinity = pd.concat([Dalat_scores, Vicinity_scores], ignore_index=True)
```

```
hypothesise_UVs_total_scores = (
    combine_score_Dalat_vicinity
    .groupby(["Source", "Village"])["Score"]
    .sum(min_count=1)   # ignore NaN
    .reset_index()
)
```

5.1.4. GUV_UVC

```
# Group by Category_EN
GUV_UVC_Category_EN = (
    village_GUV_meeting_UVC
    .groupby(["Village", "Category_EN"])
    .size()
    .reset_index(name="Count")
)
```

```
GUV_UVC_Category_EN.head()
```

Show hidden output

```
# Group by designation_EN
GUV_UVC_Criteria_designation_EN = (
    village_GUV_meeting_UVC
    .groupby(["Village", "Criteria designation_EN"])
    .size()
    .reset_index(name="Count")
)
```

```
GUV_UVC_Criteria_designation_EN.head(8)
```

Show hidden output

Next steps: Generate code with GUV_UVC_Criteria_designation_EN | New interactive sheet

```
GUV_UVC_total_scores = (
    GUV_UVC_scores
    .groupby("Village", as_index=False)["Score"]
    .sum(min_count=1)
)
GUV_UVC_total_scores
```

Show hidden output

5.1.5. GUV_total

```
# Group by Category_EN
GUV_total_Category_EN = (
    village_GUV_meeting_total
    .groupby(["Village", "Category_EN"])
    .size()
    .reset_index(name="Count")
)
```

```
GUV_total_Category_EN.head(10)
```

Show hidden output

Next steps: Generate code with GUV_total_Category_EN | New interactive sheet

```
# Group by designation_EN
GUV_total_Criteria_designation_EN = (
    village_GUV_meeting_total
    .groupby(["Village", "Criteria designation_EN"])
    .size()
    .reset_index(name="Count")
)
```

```
GUV_total_Criteria_designation_EN.head(10)
```

Show hidden output

Next steps: Generate code with GUV_total_Criteria_designation_EN | New interactive sheet

```
GUV_total_total_scores = (
    GUV_total_scores
    .groupby("Village", as_index=False)["Score"]
    .sum(min_count=1)
)
GUV_total_total_scores
```

Show hidden output

5.1.6. Super village

```
# --- 1: Da loc village
# Take data of Da loc village from Da Lat dataframe
DaLoc = df_dalat[
    (df_dalat["Survey result"] == "true") &
    (df_dalat["Village"] == "Da Loc") &
    (df_dalat["ID_new"].isin(df_OVs["ID_new"]))
].copy()

# Take the necessary column for Da Loc village
DaLoc = DaLoc.merge(
    df_OVs[["ID_new", "Category_EN", "Sub_category_EN", "Criteria designation_EN"]],
    on="ID_new",
    how="left"
)

DaLoc["Village"] = "The Combined Village"

# ---2: GUV
# Similarly
GUV = df_GUV[
    (df_GUV['Survey result'] == "true") &
    (df_GUV["ID_new"].isin(df_OVs["ID_new"]))
].copy()

GUV = GUV.merge(
    df_OVs[["ID_new", "Category_EN", "Sub_category_EN", "Criteria designation_EN"]],
    on="ID_new",
    how="left"
)

GUV["Village"] = "The Combined Village"

# --- 3: Union 2 tables ---
CombinedTable = pd.concat([DaLoc, GUV], ignore_index=True)

# --- 4. Groupby by ID_new ---
Combined_village = (
    CombinedTable.groupby("ID_new", as_index=False)
    .agg({
        "Village": "max",
        "Category_EN": "max",
        "Sub_category_EN": "max",
        "Criteria designation_EN": "max"
    })
)
```

```
display(Combined_village.head(5))
display(Combined_village.tail(5))
```

Show hidden output

```
# Group by Category_EN
Combined_village_Category_EN = (
    Combined_village
    .groupby(["Village", "Category_EN"])
    .size()
    .reset_index(name="Count")
)
```

```
Combined_village_Category_EN.head(10)
```

Show hidden output

Next steps: Generate code with `Combined_village_Category_EN` New interactive sheet

```
# Group by designation_EN
Combined_village_Criteria_designation_EN = (
    Combined_village
    .groupby(["Village", "Criteria designation_EN"])
    .size()
    .reset_index(name="Count")
)
```

```
Combined_village_Criteria_designation_EN.head(10)
```

Show hidden output

Next steps: Generate code with `Combined_village_Criteria_designation_EN` New interactive sheet

```
# Scoring Combined Village:
Combined_village_scores = pd.concat([
    compute_scores(v, criteria_weight, Combined_village, df_OVs)
    for v in Combined_village["Village"].unique()
])

Combined_village_total_scores = (
    Combined_village_scores
```

```
    .groupby("Village", as_index=False)["Score"]
    .sum(min_count=1)
)
```

```
display(Combined_village_scores.head(10))
display(Combined_village_total_scores.head(10))
```

Show hidden output

5.2. Visualize by plotly library

```
import plotly.express as px
```

By topic

```
# Stacked bar chart, distinguish by Source
fig = px.bar(
    combined_Category_EN,
    y="Village",
    x="Count",
    color="Category_EN",
    orientation="h",
    barmode="stack",
    color_discrete_sequence=px.colors.sequential.Blues  # monochromatic scale from dark to light
)

# font, size
fig.update_layout(
    xaxis_title="Number of OVs achieved by literature topic",
    yaxis_title="",
    font=dict(
        family="Times New Roman",
        size=8
    ),
    width=397,   # 1/2 width of A4
    height=1123,  # full height of A4
    legend=dict(
        orientation="h",   # horizontal
        y=-0.05,            # under the chart
        x=0.5,             # center
        xanchor="center",
        title_text=""
    ),
    plot_bgcolor="white",
    paper_bgcolor="white",
    xaxis=dict(
        showline=True,
        linewidth=0.5,
        linecolor="black",
        mirror=True,
        showgrid=True,          # projection line
        gridcolor="lightgrey",  # projection line's color
        gridwidth=0.5
    ),
    yaxis=dict(
        showline=True,
        linewidth=0.5,
        linecolor="black",
        mirror=True,
        categoryorder="array",
        categoryarray=combined_Category_EN.groupby("Village")["Count"].sum().sort_values(ascending=False).index
    )
)

fig.show()
```

Show hidden output

By Criteria

```
# Stacked bar chart, distinguish by Source
fig = px.bar(
    combined_Criteria_designation_EN,
    y="Village",
    x="Count",
    color="Criteria designation_EN",
    orientation="h",
    barmode="stack",
    color_discrete_sequence=px.colors.sequential.Blues  # monochromatic scale from dark to light
)

# font, size
fig.update_layout(
    xaxis_title="Number of OVs achieved by Criteria",
    yaxis_title="",
    font=dict(
        family="Times New Roman",
        size=8
    ),
    width=397,   # 1/2 width of A4
    height=1123,  # full height of A4
    legend=dict(
```

```
        orientation="h",   # horizontal
        y=-0.05,            # under the chart
        x=0.5,             # center
        xanchor="center",
        title_text=""
    ),
    plot_bgcolor="white",
    paper_bgcolor="white",
    xaxis=dict(
        showline=True,
        linewidth=0.5,
        linecolor="black",
        mirror=True,
        showgrid=True,          # projection line
        gridcolor="lightgrey",  # projection line's color
        gridwidth=0.5
    ),
    yaxis=dict(
        showline=True,
        linewidth=0.5,
        linecolor="black",
        mirror=True,
        categoryorder="array",
        categoryarray=combined_Criteria_designation_EN.groupby("Village")["Count"].sum().sort_values(ascending=False).index
    )
)

fig.show()
```

Show hidden output

5.3. Visualize by matplotlib library

```
import matplotlib.pyplot as plt
```

5.2.1. Number of OVs meeting - by literature topic

```
# Sort Village alphabetically
village_order_Category_EN = sorted(combined_Category_EN["Village"].unique())
combined_Category_EN["Village"] = pd.Categorical(
    combined_Category_EN["Village"], categories=village_order_Category_EN, ordered=True
)

# Pivot the data to form a stacked bar
pivot_table_Category_EN = combined_Category_EN.pivot_table(
    index="Village", columns="Category_EN", values="Count", aggfunc='sum', fill_value=0
)

# Monochromatic color scheme from dark to light blue
colors = plt.cm.Greys(np.linspace(0.8, 0.15, len(pivot_table_Category_EN.columns)))

# --- Convert font to serif ---
plt.rcParams["font.family"] = "serif"

# --- Draw chart ---
fig, ax = plt.subplots(figsize=(4, 11.2))  # inch, equivalent to 1/2 A4 x full A4

bottom = np.zeros(len(pivot_table_Category_EN))
villages = pivot_table_Category_EN.index.tolist()

for i, col in enumerate(pivot_table_Category_EN.columns):
    ax.barh(villages, pivot_table_Category_EN[col], left=bottom, color=colors[i], label=col)
    bottom += pivot_table_Category_EN[col].values

# Refine
ax.set_xlabel("Number of OVs achieved by literature topic", fontsize=10)
ax.set_ylabel("")
ax.tick_params(axis='y', labelsize=8)
ax.tick_params(axis='x', labelsize=8)
ax.set_facecolor("white")
ax.grid(axis='x', color='lightgrey', linewidth=0.5)
ax.spines['top'].set_linewidth(0.5)
ax.spines['right'].set_linewidth(0.5)
ax.spines['bottom'].set_linewidth(0.5)
ax.spines['left'].set_linewidth(0.5)
ax.legend(loc='lower center', bbox_to_anchor=(0.5, -0.15), ncol=1, frameon=False, fontsize=8, title='')

plt.subplots_adjust(left=0.2, right=0.95)

# --- Export file PNG and PDF high quality ---
# plt.savefig("stacked_bar_chart1_hd.png", dpi=300, bbox_inches='tight')

plt.show()
```

Show hidden output

5.2.2. Number of OVs meeting - by Criteria

```
# Calculate the total Count by Village to sort in descending order
village_order = sorted(combined_Criteria_designation_EN["Village"].unique())
combined_Criteria_designation_EN["Village"] = pd.Categorical(
    combined_Criteria_designation_EN["Village"], categories=village_order, ordered=True
```

```
)

# Pivot data to form a stacked bar
pivot_table = combined_Criteria_designation_EN.pivot_table(
    index="Village", columns="Criteria designation_EN", values="Count", aggfunc='sum', fill_value=0
)

# Monochromatic color from dark blue to light blue.
colors = plt.cm.Greys(np.linspace(0.9, 0.15, len(pivot_table.columns)))

# --- Convert font to serif ---
plt.rcParams["font.family"] = "serif"

# --- Draw chart ---
fig, ax = plt.subplots(figsize=(4, 11.2))  # inch, equivalent to 1/2 A4 x full A4

bottom = np.zeros(len(pivot_table))
villages = pivot_table.index.tolist()

for i, col in enumerate(pivot_table.columns):
    ax.barh(villages, pivot_table[col], left=bottom, color=colors[i], label=col)
    bottom += pivot_table[col].values

# Refine
ax.set_xlabel("Number of OVs achieved by Criteria", fontsize=10)
ax.set_ylabel("")
ax.tick_params(axis='y', labelsize=8)
ax.tick_params(axis='x', labelsize=8)
ax.set_facecolor("white")
ax.grid(axis='x', color='lightgrey', linewidth=0.5)
ax.spines['top'].set_linewidth(0.5)
ax.spines['right'].set_linewidth(0.5)
ax.spines['bottom'].set_linewidth(0.5)
ax.spines['left'].set_linewidth(0.5)
ax.legend(loc='lower center', bbox_to_anchor=(0.5, -0.15), ncol=2, frameon=False, fontsize=8, title='')

plt.subplots_adjust(left=0.2, right=0.95)

# --- Export file PNG and PDF high quality ---
# plt.savefig("stacked_bar_chart2_hd.png", dpi=300, bbox_inches='tight')

plt.show()
```

Show hidden output

5.2.3. Total scores of the villages

```
plt.figure(figsize=(10, 6))

# Draw line chart for each Source
for source, data in hypothesise_UVs_total_scores.groupby("Source"):
    plt.plot(
        data["Village"],
        data["Score"],
        marker="o",
        label=source
    )

plt.xticks(rotation=90, fontsize=8)
plt.xlabel("Village")
plt.ylabel("Total Score")
plt.title("Total Score by Village (Da Lat vs Vicinity)")
plt.legend()
plt.grid(True, linestyle="--", alpha=0.6)

plt.tight_layout()
plt.show()
```

Show hidden output

```
# villages and their y positions (in the order prepared in pivot_table.index)
villages = pivot_table.index.tolist()
y_pos = np.arange(len(villages))

# --- Stacked bar using y_pos for easier synchronization with line ---
fig, ax1 = plt.subplots(figsize=(4, 11.2))

bottom = np.zeros(len(pivot_table))
for i, col in enumerate(pivot_table.columns):
    vals = pivot_table[col].values
    ax1.barh(y_pos, vals, left=bottom, color=colors[i], label=col)
    bottom = bottom + vals

# Assign the Y label with the village name (in the correct order)
ax1.set_yticks(y_pos)
ax1.set_yticklabels(villages)

# Format bar chart
ax1.set_xlabel("Number of OVs achieved by Criteria", fontsize=10)
ax1.tick_params(axis='y', labelsize=8)
ax1.tick_params(axis='x', labelsize=8)
ax1.set_facecolor("white")
ax1.grid(axis='x', color='lightgrey', linewidth=0.5)
for spine in ['top','right','bottom','left']:
    ax1.spines[spine].set_linewidth(0.5)
```

```
# --- Prepare the Score series in the correct village order ---
# If hypothesis_UVs_total_scores has multiple sources (Da Lat/Vicinity), we will summarize:
scores_series = hypothesise_UVs_total_scores.groupby("Village")["Score"].sum(min_count=1)
# Reindex by villages to ensure the order matches the bar; if missing -> NaN
scores_aligned = scores_series.reindex(villages)

# --- Line chart (Score) on secondary x-axis ---
ax2 = ax1.twiny()
x = scores_aligned.values  # aligned to villages order

# If there is NaN, draw each segment that does not connect through NaN
mask = ~np.isnan(x)
if mask.any():
    # Find consecutive True segments to draw without connecting through NaN
    idx = np.where(mask)[0]
    splits = np.split(idx, np.where(np.diff(idx) != 1)[0] + 1)
    for seg in splits:
        ax2.plot(x[seg], y_pos[seg], marker="o", color="tab:blue", linestyle="--", label="Total Score")
else:
    # No score value (rare scenario)
    pass

# Synchronize the y-limit so that the line and bar coincide
ax2.set_ylim(ax1.get_ylim())

ax2.set_xlabel("Total Score (by 100)", fontsize=10)
ax2.tick_params(axis='x', labelsize=8)

# --- Legend: Keep the legend bar (multi-column) and add a legend line
bars_legend = ax1.legend(loc='lower center', bbox_to_anchor=(0.5, -0.15), ncol=2, frameon=False, fontsize=8, title='')
ax1.add_artist(bars_legend)
# Legend for line (to specify the name)
ax2.legend(loc='lower right', frameon=False, fontsize=8)

plt.subplots_adjust(left=0.2, right=0.95)

# --- Export high quality PNG ---
# plt.savefig("stacked_bar_chart3_hd.png", dpi=300, bbox_inches='tight', bbox_extra_artists=(bars_legend,))

plt.show()
```

Show hidden output

5.2.4. Green Urban Village

Evaluation with OVs of UVC

```
# Pivot data to create a stacked bar
pivot_GUV_UVC_Category_EN = GUV_UVC_Category_EN.pivot_table(
    index="Village", columns="Category_EN", values="Count", aggfunc='sum', fill_value=0
)

# Extract monochromatic colors from dark to light
colors = plt.cm.Greys(np.linspace(0.8, 0.15, len(pivot_GUV_UVC_Category_EN.columns)))

# --- Convert font to serif ---
plt.rcParams["font.family"] = "serif"

# --- Draw chart ---
fig, ax = plt.subplots(figsize=(8, 2))

bottom = np.zeros(len(pivot_GUV_UVC_Category_EN))
villages = pivot_GUV_UVC_Category_EN.index.tolist()

for i, col in enumerate(pivot_GUV_UVC_Category_EN.columns):
    ax.barh(villages, pivot_GUV_UVC_Category_EN[col], left=bottom, color=colors[i], label=col, height=0.05)
    bottom += pivot_GUV_UVC_Category_EN[col].values

# Refine
ax.set_xlabel("Number of OVs achieved by literature topic", fontsize=10)
ax.set_ylabel("")
ax.tick_params(axis='y', labelsize=8)
ax.tick_params(axis='x', labelsize=8)
ax.set_facecolor("white")
ax.grid(axis='x', color='lightgrey', linewidth=0.5)
ax.spines['top'].set_linewidth(0.5)
ax.spines['right'].set_linewidth(0.5)
ax.spines['bottom'].set_linewidth(0.5)
ax.spines['left'].set_linewidth(0.5)
ax.set_ylim(-0.3, 0.3)
ax.legend(loc='lower center', bbox_to_anchor=(0.5, -0.025), ncol=1, frameon=False, fontsize=8, title='')

plt.tight_layout()

# --- Export high quality PNG and PDF ---
# plt.savefig("stacked_bar_chart4_hd.png", dpi=300, bbox_inches='tight')

plt.show()
```

Show hidden output

```
# Pivot data to create a stacked bar
pivot_GUV_UVC_Criteria_designation_EN = GUV_UVC_Criteria_designation_EN.pivot_table(
    index="Village", columns="Criteria designation_EN", values="Count", aggfunc='sum', fill_value=0
)
```

```python
# Extract monochromatic colors from dark to light
colors = plt.cm.Greys(np.linspace(0.8, 0.15, len(pivot_GUV_UVC_Criteria_designation_EN.columns)))

# --- Convert font to serif ---
plt.rcParams["font.family"] = "serif"

# --- Draw chart ---
fig, ax = plt.subplots(figsize=(8, 2))  # inch, equivalent to 1/2 A4 x full A4

bottom = np.zeros(len(pivot_GUV_UVC_Criteria_designation_EN))
villages = pivot_GUV_UVC_Criteria_designation_EN.index.tolist()

for i, col in enumerate(pivot_GUV_UVC_Criteria_designation_EN.columns):
    ax.barh(villages, pivot_GUV_UVC_Criteria_designation_EN[col], left=bottom, color=colors[i], label=col, height=0.05)
    bottom += pivot_GUV_UVC_Criteria_designation_EN[col].values

# Refine
ax.set_xlabel("Number of OVs achieved by Criteria", fontsize=10)
ax.set_ylabel("")
ax.tick_params(axis='y', labelsize=8)
ax.tick_params(axis='x', labelsize=8)
ax.set_facecolor("white")
ax.grid(axis='x', color='lightgrey', linewidth=0.5)
ax.spines['top'].set_linewidth(0.5)
ax.spines['right'].set_linewidth(0.5)
ax.spines['bottom'].set_linewidth(0.5)
ax.spines['left'].set_linewidth(0.5)
ax.set_ylim(-0.3, 0.3)
ax.legend(loc='lower center', bbox_to_anchor=(0.5, -0.6), ncol=4, frameon=False, fontsize=8, title='')

plt.subplots_adjust(left=0.2, right=0.95)

# --- Export PNG and PDF high quality ---
# plt.savefig("stacked_bar_chart4_hd.png", dpi=300, bbox_inches='tight')

plt.show()
```

Show hidden output

Evaluation with total OVs list

```python
# Pivot data to create a stacked bar
pivot_GUV_total_Category_EN = GUV_total_Category_EN.pivot_table(
    index="Village", columns="Category_EN", values="Count", aggfunc='sum', fill_value=0
)

# Extract monochromatic colors from dark to light
colors = plt.cm.Greys(np.linspace(0.8, 0.15, len(pivot_GUV_total_Category_EN.columns)))

# --- Convert font to serif ---
plt.rcParams["font.family"] = "serif"

# --- Draw chart ---
fig, ax = plt.subplots(figsize=(8, 2))

bottom = np.zeros(len(pivot_GUV_total_Category_EN))
villages = pivot_GUV_total_Category_EN.index.tolist()

for i, col in enumerate(pivot_GUV_total_Category_EN.columns):
    ax.barh(villages, pivot_GUV_total_Category_EN[col], left=bottom, color=colors[i], label=col, height=0.05)
    bottom += pivot_GUV_total_Category_EN[col].values

# Refine
ax.set_xlabel("Number of OVs achieved by literature topic", fontsize=10)
ax.set_ylabel("")
ax.tick_params(axis='y', labelsize=8)
ax.tick_params(axis='x', labelsize=8)
ax.set_facecolor("white")
ax.grid(axis='x', color='lightgrey', linewidth=0.5)
ax.spines['top'].set_linewidth(0.5)
ax.spines['right'].set_linewidth(0.5)
ax.spines['bottom'].set_linewidth(0.5)
ax.spines['left'].set_linewidth(0.5)
ax.set_ylim(-0.3, 0.3)
ax.legend(loc='lower center', bbox_to_anchor=(0.5, -0.025), ncol=2, frameon=False, fontsize=8, title='')

plt.tight_layout()

# --- Export high quality PNG and PDF ---
# plt.savefig("stacked_bar_chart4_hd.png", dpi=300, bbox_inches='tight')

plt.show()
```

Show hidden output

```python
# Pivot data to create a stacked bar
pivot_GUV_total_Criteria_designation_EN = GUV_total_Criteria_designation_EN.pivot_table(
    index="Village", columns="Criteria designation_EN", values="Count", aggfunc='sum', fill_value=0
)

# Extract monochromatic colors from dark to light
colors = plt.cm.Greys(np.linspace(0.8, 0.15, len(pivot_GUV_total_Criteria_designation_EN.columns)))

# --- Convert font to serif ---
plt.rcParams["font.family"] = "serif"
```

```
# --- Draw chart ---
fig, ax = plt.subplots(figsize=(8, 2))  # inch, Equivalent to 1/2 A4 x full A4

bottom = np.zeros(len(pivot_GUV_total_Criteria_designation_EN))
villages = pivot_GUV_total_Criteria_designation_EN.index.tolist()

for i, col in enumerate(pivot_GUV_total_Criteria_designation_EN.columns):
    ax.barh(villages, pivot_GUV_total_Criteria_designation_EN[col], left=bottom, color=colors[i], label=col, height=0.05)
    bottom += pivot_GUV_total_Criteria_designation_EN[col].values

# Refine
ax.set_xlabel("Number of OVs achieved by Criteria", fontsize=10)
ax.set_ylabel("")
ax.tick_params(axis='y', labelsize=8)
ax.tick_params(axis='x', labelsize=8)
ax.set_facecolor("white")
ax.grid(axis='x', color='lightgrey', linewidth=0.5)
ax.spines['top'].set_linewidth(0.5)
ax.spines['right'].set_linewidth(0.5)
ax.spines['bottom'].set_linewidth(0.5)
ax.spines['left'].set_linewidth(0.5)
ax.set_ylim(-0.3, 0.3)
ax.legend(loc='lower center', bbox_to_anchor=(0.5, -0.6), ncol=4, frameon=False, fontsize=8, title='')

plt.subplots_adjust(left=0.2, right=0.95)

# --- Export PNG and PDF ---
# plt.savefig("stacked_bar_chart5_hd.png", dpi=300, bbox_inches='tight')

plt.show()
```

Show hidden output

5.2.5. Super Village

```
# Pivot data to create a stacked bar
pivot_Combined_village_Category_EN = Combined_village_Category_EN.pivot_table(
    index="Village", columns="Category_EN", values="Count", aggfunc='sum', fill_value=0
)

# Extract monochromatic colors from dark to light
colors = plt.cm.Greys(np.linspace(0.8, 0.15, len(pivot_Combined_village_Category_EN.columns)))

# --- Convert font to serif ---
plt.rcParams["font.family"] = "serif"

# --- Draw chart ---
fig, ax = plt.subplots(figsize=(8, 2))

bottom = np.zeros(len(pivot_Combined_village_Category_EN))
villages = pivot_Combined_village_Category_EN.index.tolist()

for i, col in enumerate(pivot_Combined_village_Category_EN.columns):
    ax.barh(villages, pivot_Combined_village_Category_EN[col], left=bottom, color=colors[i], label=col, height=0.05)
    bottom += pivot_Combined_village_Category_EN[col].values

# Refine
ax.set_xlabel("Number of OVs achieved by literature topic", fontsize=10)
ax.set_ylabel("")
ax.tick_params(axis='y', labelsize=8)
ax.tick_params(axis='x', labelsize=8)
ax.set_facecolor("white")
ax.grid(axis='x', color='lightgrey', linewidth=0.5)
ax.spines['top'].set_linewidth(0.5)
ax.spines['right'].set_linewidth(0.5)
ax.spines['bottom'].set_linewidth(0.5)
ax.spines['left'].set_linewidth(0.5)
ax.set_ylim(-0.3, 0.3)
ax.legend(loc='lower center', bbox_to_anchor=(0.5, -0.6), ncol=4, frameon=False, fontsize=8, title='')

plt.subplots_adjust(left=0.2, right=0.95)

# --- Export PNG and PDF ---
plt.savefig("stacked_bar_chart6_hd.png", dpi=300, bbox_inches='tight')

plt.show()
```

Show hidden output

```
# Pivot data to create a stacked bar
pivot_Combined_village_Criteria_designation_EN = Combined_village_Criteria_designation_EN.pivot_table(
    index="Village", columns="Criteria designation_EN", values="Count", aggfunc='sum', fill_value=0
)

# Extract monochromatic colors from dark to light
colors = plt.cm.Greys(np.linspace(0.8, 0.15, len(pivot_Combined_village_Criteria_designation_EN.columns)))

# --- Convert font to serif ---
plt.rcParams["font.family"] = "serif"

# --- Draw chart ---
fig, ax = plt.subplots(figsize=(8, 2))

bottom = np.zeros(len(pivot_Combined_village_Criteria_designation_EN))
villages = pivot_Combined_village_Criteria_designation_EN.index.tolist()
```

```
for i, col in enumerate(pivot_Combined_village_Criteria_designation_EN.columns):
    ax.barh(villages, pivot_Combined_village_Criteria_designation_EN[col], left=bottom, color=colors[i], label=col, height=0.05)
    bottom += pivot_Combined_village_Criteria_designation_EN[col].values

# Refine
ax.set_xlabel("Number of OVs achieved by Criteria", fontsize=10)
ax.set_ylabel("")
ax.tick_params(axis='y', labelsize=8)
ax.tick_params(axis='x', labelsize=8)
ax.set_facecolor("white")
ax.grid(axis='x', color='lightgrey', linewidth=0.5)
ax.spines['top'].set_linewidth(0.5)
ax.spines['right'].set_linewidth(0.5)
ax.spines['bottom'].set_linewidth(0.5)
ax.spines['left'].set_linewidth(0.5)
ax.set_ylim(-0.3, 0.3)
ax.legend(loc='lower center', bbox_to_anchor=(0.5, -0.6), ncol=4, frameon=False, fontsize=8, title='')

plt.subplots_adjust(left=0.2, right=0.95)

# --- Export PNG and PDF ---
plt.savefig("stacked_bar_chart7_hd.png", dpi=300, bbox_inches='tight')

plt.show()
```

Show hidden output
