# Supplementary material for: (Re)defining urban villages and their potential in sustaining local authenticity: A case study of Da Lat, Viet Nam
Source: PLoS One. 2026 Apr 3;21(4):e0345741. doi: 10.1371/journal.pone.0345741 (PMC13048443; doi:10.1371/journal.pone.0345741)
Supplement: S1 Table — (DOCX) [file pone.0345741.s011.docx]

| Literature topic | Criteria | Sector | No. | ID | Description |
| --- | --- | --- | --- | --- | --- |
| Ethnic enclave | **Community and Social Structures** | Social feature | 1 | X_EE_03 | It is a tightly knit community (between families and the residential community). |
|  | **Cultural Preservation** | Social feature | 2 | X_EE_07 | Residents have a positive awareness of maintaining their identity. |
|  | **Social and Spatial Cohesion** | Social feature | 3 | X_EE_02 | Urban village residents have adapted to the urban environment. |
| Indigenous villages in urban areas - Central Highlands VN | **Addressing Resident Needs** | Environmental feature | 4 | M_ViC_TN_025 | Many families still maintain traditional stilt houses but have built an additional ground-level house next to them. |
|  | **Community and Social Structures** | Economic feature | 5 | K_ViC_TN_02 | The practice of subdividing and selling land to Kinh people has become widespread |
|  | **Community and Social Structures** | Institutional and governance feature | 6 | TQ_ViC_TN_06 | The authority of the Village Elders’ Council remains especially important to the villagers. |
|  | **Cultural Preservation** | Environmental feature | 7 | M_ViC_TN_011 | Tangible cultural heritage values: topography, landscape, and natural environment. |
|  |  |  | 8 | M_ViC_TN_012 | Tangible cultural heritage values: architectural structures (communal houses, longhouses, residential houses, granaries, stilt houses, village gates, tomb houses, and grave statues, etc.). |
|  |  |  | 9 | M_ViC_TN_013 | Tangible cultural heritage values: cultural and religious institutions (communal house areas, tomb house areas, ritual offerings to deities, jars, gongs, backpacks, bows and arrows, machetes, various musical instruments). |
|  |  |  | 10 | M_ViC_TN_06 | The indigenous village was established long ago, predating the formation of the urban area. |
|  |  | Institutional and governance feature | 11 | TQ_ViC_TN_07 | The local authorities are concerned with restoring and maintaining the communal house (nhà rông), seeing it as the soul of the traditional village. |
|  |  | Social feature | 12 | X_ViC_TN_01 | Each indigenous ethnic group has distinctive cultural traditions, with many customs and folk festivals. |
|  |  |  | 13 | X_ViC_TN_02 | Traditional customs often have intangible cultural heritage value. |
|  |  |  | 14 | X_ViC_TN_03 | Intangible cultural heritage value: gong cultural space, gong art. |
|  |  |  | 15 | X_ViC_TN_04 | Intangible cultural heritage value: Central Highlands epics—epic culture, long poems, various forms of folk culture such as literature, folk songs, folk music, festivals, and customs. |
|  |  |  | 16 | X_ViC_TN_05 | Intangible cultural heritage value: culinary cultural identity. |
|  |  |  | 17 | X_ViC_TN_06 | Intangible cultural heritage value: household items, production tools, and traditional crafts. |
|  |  |  | 18 | X_ViC_TN_08 | Intangible cultural heritage value: Central Highlands traditional culture reflected in the community’s egalitarian way of life, along with the role of the village elder. |
|  |  |  | 19 | X_ViC_TN_09 | Intangible cultural heritage value: Central Highlands traditional culture reflected in the sculptural art of mortuary statues, communal houses (nhà rông), textile patterns, and architecture. |
|  | **Migrant and Ethnic Integration** | Social feature | 20 | X_ViC_TN_010 | The influence of the Kinh people, such as the concept of ground-floor houses and solid construction, has become widespread. |
|  | **Social and Spatial Cohesion** | Environmental feature | 21 | M_ViC_TN_020 | In the case of villages of ethnic groups belonging to the Mon-Khmer language family (Bahnar, Xơ Đăng, Giẻ Triêng, Cơ Ho, Mạ, etc.), they reside in the form of clustered residential communities (each village represents a single settlement where all village families gather). |
|  |  |  | 22 | M_ViC_TN_030 | There is a festival courtyard. |
|  |  |  | 23 | M_ViC_TN_043 | Characteristic public space of indigenous villages: Gong culture space. |
|  |  |  | 24 | M_ViC_TN_045 | Characteristic public space of indigenous villages: The communal house (nhà rông) is a space where religious rituals and important village affairs take place. |
|  |  |  | 25 | M_ViC_TN_047 | Characteristic public space of indigenous villages: The festival courtyard is a space where religious rituals and important village affairs take place. |
|  |  |  | 26 | M_ViC_TN_05 | The village is currently located in a suburban area. |
|  |  |  | 27 | M_ViC_TN_054 | There is a close relationship between people and nature. |
|  |  |  | 28 | M_ViC_TN_09 | Each indigenous ethnic group has its own traditions for building villages and housing. |
|  |  | Institutional and governance feature | 29 | TQ_ViC_TN_03 | Planning and construction of traditional villages within the city almost lack clear orientation. |
|  |  |  | 30 | TQ_ViC_TN_08 | For villages without a traditional communal house, the local authority has proposed building a community activity house. |
|  | **Sustainable Economic Development** | Economic feature | 31 | K_ViC_TN_01 | A few villages with potential for tourism services have only made small-scale investments, such as basic visitor facilities, souvenir shops, and simple cultural performances |
| Traditional villages - Northern & Central VN | **Community and Social Structures** | Institutional and governance feature | 32 | TQ_BT_019 | Within the village, there are hamlets (a village/commune can have many hamlets, which are clusters of houses adjacent to each other, with one main road and houses on both sides). |
|  |  |  | 33 | TQ_BT_020 | Within the village, there are alleys (an alley is a subdivision of a hamlet; a hamlet can have many alleys, which are narrow lanes in the hamlet containing adjacent families). |
|  |  |  | 34 | TQ_BT_03 | Rural organization is based on residential areas: hamlets and villages. Village members are bonded by production relations, mutual help, and solidarity on the basis of respect and equality. |
|  |  | Social feature | 35 | X_BT_017 | Worship of the Village Deity (Thành hoàng) is a communal activity, with the entire community participating in the rituals. |
|  |  |  | 36 | X_BT_04 | The community and autonomy in the village have both positive and negative consequences. |
|  |  |  | 37 | X_BT_07 | Kinship ties exist strongly between village members. |
|  | **Cultural Preservation** | Environmental feature | 38 | M_BT_03 | The village temple serves as the cultural center |
|  |  |  | 39 | M_BT_04 | The village temple also serves as the spiritual and religious center |
|  |  | Social feature | 40 | X_BT_011 | The village has cultural and entertainment clubs. |
|  |  |  | 41 | X_BT_08 | The village festival (hội làng) is a typical cultural activity — a regular, fixed-time event that mobilizes and directly involves all community members and embodies cultural significance. |
|  | **Migrant and Ethnic Integration** | Social feature | 42 | X_BT_06 | Residents have “migrated” the village lifestyle to their new homes and livelihoods. |
|  | **Social and Spatial Cohesion** | Environmental feature | 43 | M_BT_023 | A village or hamlet may consist of several smaller settlements (xóm) |
|  |  |  | 44 | M_BT_024 | Residents build their homes along both sides of the road |
|  | **Sustainable Economic Development** | Economic feature | 45 | K_BT_04 | Contributing to the preservation and development of handicrafts |
| Traditional villages - Northern VN | **Social and Spatial Cohesion** | Environmental feature | 46 | M_B_02 | In semi-mountainous areas, villages are often built in low-lying areas near water sources, surrounded by hills and mountains |
| Urban village concept | **Addressing Resident Needs** | Economic feature | 47 | K_C_01 | Prioritizing mixed land use; integrating residential, service, commercial, recreational, and communal functions to the greatest extent possible |
|  |  |  | 48 | K_C_03 | The prioritization of mixed-use development is practiced at the levels of neighborhoods, street blocks, and buildings |
|  |  | Environmental feature | 49 | M_C_030 | Ensure feasible, high-quality public transport to reduce reliance on private cars |
|  |  |  | 50 | M_C_031 | Encourage walking and cycling by providing good routes and infrastructure, while creating spaces that are equitably shared by all users |
|  |  |  | 51 | M_C_039 | New buildings must be adaptable to modern lifestyles. |
|  |  |  | 52 | M_C_040 | The safety and mobility of children should be central to planning and design. |
|  |  |  | 53 | M_C_042 | Respect the value of schools and kindergartens as essential local amenities. |
|  |  |  | 54 | M_C_08 | Provide a pedestrian-friendly environment conducive to walking. |
|  |  |  | 55 | M_C_09 | Include a primary school. |
|  |  |  | 56 | M_EX_1 | Invest in transport infrastructure amenities such as parking lots. |
|  |  |  | 57 | M_EX_4 | Develop nighttime service systems to meet residents’ needs and attract tourists. |
|  |  |  | 58 | M_EX_8 | Planning criteria related to healthcare and technology should be added, ensuring a proper medical infrastructure system and the application of technology in production and services. |
|  |  | Institutional and governance feature | 59 | TQ_C_03 | Consider the perspectives, aspirations, and needs of local stakeholders. |
|  |  | Social feature | 60 | X_C_05 | It is necessary to address broader social goals and concerns. |
|  |  |  | 61 | X_C_07 | Provide children access to educational, social, and employment networks. |
|  | **Community and Social Structures** | Environmental feature | 62 | M_C_038 | Ensure security and foster a strong sense of respect for the law within the community. |
|  |  | Institutional and governance feature | 63 | TQ_C_01 | It is sustainably managed by local residents. |
|  |  |  | 64 | TQ_C_06 | Leverage the ability to build mutual support networks within the local community. |
|  |  |  | 65 | TQ_EX_4 | Establish a multi-tier urban village management model combining state administrative management and community self-management. |
|  |  |  | 66 | TQ_EX_5 | Develop a flexible urban village management mechanism that both complies with the general legal framework and maintains specific local customs suited to the local identity. |
|  |  |  | 67 | TQ_EX_6 | Develop a flexible urban management mechanism that both ensures compliance with the general legal framework and maintains specific local customs suited to the local identity. |
|  |  | Social feature | 68 | X_C_010 | Respect and revive faith in the life of the urban village. |
|  |  |  | 69 | X_C_02 | The society is integrated and has community consultation. |
|  |  |  | 70 | X_C_04 | The society achieves a certain degree of autonomy. |
|  |  |  | 71 | X_C_08 | Recognize the important social role of primary schools in residential life. |
|  |  |  | 72 | X_C_09 | Integrate formal education with the daily life of the community. |
|  | **Cultural Preservation** | Environmental feature | 73 | M_C_013 | Local identity serves as the foundation for establishing a typical architectural style |
|  |  |  | 74 | M_C_023 | Reflect traditional and local characteristics in the spatial structure of urban villages |
|  |  |  | 75 | M_C_032 | Respect and enhance the unique character of each place within the village, while accepting non-uniformity with national standards |
|  |  | Social feature | 76 | X_C_011 | Preserve, conserve, inherit, and promote traditional cultural values as intangible heritage. |
|  |  |  | 77 | X_EX_2 | Preserve and develop the indigenous language, encouraging its use in community life to maintain cultural identity. |
|  |  |  | 78 | X_EX_5 | Preserve tangible and intangible cultural heritage, maintaining architecturally valuable historical and cultural structures in urban village development planning. |
|  | **Environmental and Ecological Integrity** | Environmental feature | 79 | M_C_012 | Attention is given to traffic calming |
|  |  |  | 80 | M_C_017 | Buildings and spaces are designed to be adaptable and capable of evolving over time |
|  |  |  | 81 | M_C_019 | Architectural diversity and a sustainable urban form |
|  |  |  | 82 | M_C_046 | Encourage detached houses (with sloped roofs) and garden homes; a maximum of 3 floors; building density on each plot should range from 30–40%. Apply green building solutions and new construction materials, gradually implemented for renovation projects according to an appropriate roadmap. |
|  |  |  | 83 | M_C_047 | Public architecture (depending on the scale of the planning solution): must reflect characteristics and forms suitable for the "Green Urban Village" model. |
|  |  |  | 84 | M_C_048 | Technical infrastructure systems must be fully invested, connected, and operated synchronously according to investment phases, using environmentally friendly equipment and technology lines. |
|  |  |  | 85 | M_C_049 | Social infrastructure systems should promote green lifestyles and sustainable consumption. Apply urban planning and design standards to non-agricultural areas. |
|  |  |  | 86 | M_C_050 | Collect and recycle household waste; conserve electricity. |
|  |  |  | 87 | M_C_051 | Encourage the use of natural and renewable energy such as solar and wind power. |
|  |  |  | 88 | M_C_052 | A green environment (artificial or natural) should be environmentally friendly and harmonious, balancing conservation with development. |
|  |  |  | 89 | M_C_053 | Address broader goals and concerns regarding environmental issues. |
|  |  |  | 90 | M_EX_10 | Apply renewable energy. |
|  |  |  | 91 | M_EX_11 | Improve microclimate, design green spaces, water bodies, and natural ventilation systems to create a cool and fresh living environment. |
|  |  |  | 92 | M_EX_12 | Conserve and develop urban forests, maintain green areas within the urban village. |
|  |  |  | 93 | M_EX_13 | Address shortcomings in environmental pollution treatment. |
|  |  |  | 94 | M_EX_14 | Plan urban village green spaces using native species (e.g., Da Lat pines) and agricultural greenery (flowers, vegetables) to optimize carbon absorption and regulate the microclimate. |
|  |  |  | 95 | M_EX_15 | Integrate ecological drainage systems, including regulating lakes, vegetated drainage canals, and constructed wetlands, to reduce pressure on urban drainage and naturally treat stormwater. Design water flow according to Da Lat's hilly terrain to minimize soil erosion and protect topographical integrity. |
|  |  |  | 96 | M_EX_16 | Develop a multi-layered green space network with pedestrian and cycling routes connecting residential areas, agricultural production zones, and natural forests, forming a sustainable ecological corridor. |
|  |  |  | 97 | M_EX_17 | Enhance climate change adaptation through the design of surface water infiltration systems to minimize risks of flooding and landslides. |
|  |  |  | 98 | M_EX_18 | Control the rate of concrete coverage, limit the use of retaining walls that disrupt the village's natural landscape, and encourage sustainable engineering solutions to protect terrain. |
|  |  |  | 99 | M_EX_19 | Plan buildings according to green architecture criteria, ensuring the use of environmentally friendly materials, energy efficiency, and full utilization of natural conditions. |
|  |  |  | 100 | M_EX_20 | Maintain and conserve natural landscapes, preserving the natural terrain to ensure harmony between the built environment and the native ecosystem. |
|  |  |  | 101 | M_EX_23 | Construct according to green standards. |
|  |  |  | 102 | M_EX_6 | Agricultural production areas require in-depth study and should be designated as separate zones, isolated from residential areas to protect environmental quality. |
|  |  |  | 103 | M_EX_9 | Environmental management strategies should include effective solutions for waste and wastewater treatment. |
|  |  | Institutional and governance feature | 104 | TQ_C_08 | Apply environmentally friendly management, investment monitoring, and post-investment supervision processes to the entire “Green Urban Village.” |
|  | **Migrant and Ethnic Integration** | Social feature | 105 | X_EX_3 | Incorporate indigenous culture into urban spaces by organizing activities such as festivals, markets, and common gathering places, facilitating interaction and integration between native residents and newcomers. |
|  | **Social and Spatial Cohesion** | Economic feature | 106 | K_EX_10 | Public open spaces and pedestrian traffic areas should be utilized for organizing night markets, flower exhibitions, or outdoor cultural events, combined with the sale of agricultural products and tourism experiences |
|  |  |  | 107 | K_EX_2 | Land use planning criteria need to be developed |
|  |  | Environmental feature | 108 | M_C_010 | Emphasis is placed on open space designs |
|  |  |  | 109 | M_C_011 | A connected street network is prioritized |
|  |  |  | 110 | M_C_014 | Attention is given to urban design elements such as architectural landmarks, street corners, building lines, visual recognition, visual impressions, and enclosure |
|  |  |  | 111 | M_C_016 | Continuity and pedestrian-friendliness are emphasized, with efforts to minimize dead-end alleys |
|  |  |  | 112 | M_C_018 | Bringing life to buildings and the spaces in front of them |
|  |  |  | 113 | M_C_02 | An appropriate density to support public transportation and other services |
|  |  |  | 114 | M_C_021 | High standards of urban design |
|  |  |  | 115 | M_C_022 | Emphasis on accessibility to public open spaces and green areas |
|  |  |  | 116 | M_C_024 | High-quality and well-connected public spaces |
|  |  |  | 117 | M_C_025 | Connection to nearby urban areas through an optimized public transportation system |
|  |  |  | 118 | M_C_026 | Enhance connectivity within and between urban villages, as well as with the urban center |
|  |  |  | 119 | M_C_028 | Planned according to human scale and pace of life |
|  |  |  | 120 | M_C_029 | Transform commuting into a meaningful experience, not merely a means to reach a destination |
|  |  |  | 121 | M_C_033 | Ensure a balance between movement spaces and spaces for exchange and social interaction |
|  |  |  | 122 | M_C_034 | Establish key nodes in public areas, such as shops, bus stops, train stations, and parks. |
|  |  |  | 123 | M_C_035 | Promote the social role of streets and preserve local identity. |
|  |  |  | 124 | M_C_036 | Integrate the management and maintenance of public open spaces, including school playgrounds. |
|  |  |  | 125 | M_C_037 | Acknowledge that the boundary between public and private space will become more flexible in the future. |
|  |  |  | 126 | M_C_041 | Value connectivity and foster positive, joyful emotions. |
|  |  |  | 127 | M_C_05 | Include a central square. |
|  |  |  | 128 | M_C_06 | Maintain a suitable area where everything is within walking distance. |
|  |  |  | 129 | M_C_07 | Achieve "decentralized concentration," enabling people to walk or bike to points connected to high-quality public transport, from which higher-level services can be accessed. |
|  |  |  | 130 | M_EX_21 | Plan and invest in transportation infrastructure development, including appropriately located parking facilities. |
|  |  |  | 131 | M_EX_3 | Plan urban village centers to fully integrate urban amenities. |
|  |  |  | 132 | M_EX_7 | The urban village center should be oriented toward development as a multifunctional space, integrating commercial services, agricultural product hubs, accommodation, and community activities. |
|  |  | Institutional and governance feature | 133 | TQ_C_02 | Community participation in planning and implementation of plans is ensured. |
|  |  | Social feature | 134 | X_C_06 | Encourage street cultural activities |
|  | **Sustainable Economic Development** | Economic feature | 135 | K_C_013 | Producing goods through appropriate methods, linking production facilities and households within the village under modern cooperatives or other forms of collective economy |
|  |  |  | 136 | K_C_02 | Providing quality employment opportunities through effective integration of different land uses |
|  |  |  | 137 | K_C_04 | The necessity of land pooling to support urban village development |
|  |  |  | 138 | K_C_05 | Local residents benefit economically from land value appreciation following urban village planning |
|  |  |  | 139 | K_C_07 | Local trade in goods and services should aim to strengthen the local economic development network |
|  |  |  | 140 | K_C_08 | Attention should be paid to integrating economic development policies with welfare initiatives |
|  |  |  | 141 | K_C_09 | It is necessary to create a comprehensive economic development environment and diversify market options |
|  |  |  | 142 | K_EX_1 | Criteria for the development of resort and leisure services should be established |
|  |  |  | 143 | K_EX_11 | Depending on the specific conditions of the area, economic indicators should be emphasized to reflect its identity and development goals |
|  |  |  | 144 | K_EX_4 | There should be policies regarding taxation and real estate pricing |
|  |  |  | 145 | K_EX_5 | Policies on investment and investment incentives should be established |
|  |  |  | 146 | K_EX_9 | Establish a "green" brand for agricultural products (flowers, vegetables, coffee) and agrotourism, associated with sustainable standards such as chemical-free practices, water conservation, and renewable energy use |
|  |  | Institutional and governance feature | 147 | TQ_C_04 | Dialogue and design consultation must be comprehensive and extensive. |
|  |  |  | 148 | TQ_C_05 | There must be clear, long-term management and maintenance programs. |
|  |  |  | 149 | TQ_EX_1 | Address resettlement and population planning issues. |
|  |  |  | 150 | TQ_EX_3 | Organize regular public consultation sessions to gather opinions on green infrastructure and agricultural tourism. |
|  |  | Social feature | 151 | X_EX_4 | Encourage the development of traditional crafts using natural materials or agricultural by-products, combined with agritourism to create unique agricultural products. |
|  |  |  | 152 | X_EX_6 | Build urban villages with distinct identities, ensuring economic and service competitiveness to sustain development. |
|  | **Urban and Agricultural Integration** | Economic feature | 153 | K_C_011 | Promoting high-tech agricultural production oriented towards green and sustainable growth |
|  |  |  | 154 | K_C_012 | Developing agro-tourism models |
|  |  |  | 155 | K_EX_6 | The agricultural development strategy shapes the dominant economic structure of the urban village, influencing spatial distribution, production models, and residents’ livelihoods |
|  |  |  | 156 | K_EX_7 | Develop various forms of garden tourism and agricultural tourism |
|  |  |  | 157 | K_EX_8 | Integrate circular economy models into high-tech agricultural production and waste management, transforming agricultural by-products into organic fertilizers or raw materials for supporting industries |
|  |  | Environmental feature | 158 | M_C_015 | Integration of multiple functions within neighborhoods, street blocks, individual buildings, and the urban village as a whole |
|  |  |  | 159 | M_C_043 | Interspersed among urban areas are zones for the development of high-tech agricultural production combined with residential areas - these are the ‘urban villages’, offering a quality of life comparable to that of the city, and developed according to green and sustainable principles - referred to as ‘green urban villages’ |
|  |  |  | 160 | M_EX_22 | Assess the impact of greenhouse structures in urban agriculture, and consider solutions to limit their effects on microclimates and the overall landscape. |
|  |  |  | 161 | M_EX_5 | Further research is needed on residential typology, especially housing types that combine with gardens (such as small gardens, flower gardens, etc.). |
|  |  | Institutional and governance feature | 162 | TQ_C_07 | Characteristics: It is an urban zone under the general master plan; forms a high-tech agricultural production area combined with residential areas and public facilities; promotes agricultural tourism; ensures maximum protection of biodiversity, terrain, and natural-cultural landscapes of the site; reduces greenhouse gas emissions; and has synchronized infrastructure. |
| Village in the city | **Addressing Resident Needs** | Environmental feature | 163 | M_ViC_054 | Housing density has increased due to spontaneous subdivision and land partitioning. |
|  |  |  | 164 | M_ViC_055 | Many aspects of the urban village relate to sustainable forms, such as diverse housing types and densities. |
|  |  |  | 165 | M_ViC_056 | Many aspects of the urban village relate to sustainability, such as walkability and the mix of functions that support local needs. |
|  |  |  | 166 | M_ViC_057 | There is a shortage of public space. |
|  | **Community and Social Structures** | Environmental feature | 167 | M_ViC_013 | It is either a suburban village or a village located within a city center undergoing rapid urbanization. |
|  |  |  | 168 | M_ViC_042 | A variety of housing types exist. |
|  |  | Institutional and governance feature | 169 | TQ_ViC_010 | The urban village is under the management of the city authorities. |
|  |  |  | 170 | TQ_ViC_06 | There is a neighborhood or village management committee. |
|  |  | Social feature | 171 | X_ViC_029 | Demonstrates close social relationships, providing a traditional and intimate support network for mutual assistance, along with community bonding and solidarity. |
|  |  |  | 172 | X_ViC_035 | Most urban villages still preserve core values that help bind the community through communal activities organized during village festivals. |
|  |  |  | 173 | X_ViC_05 | Undergoing many transformations in identity. |
|  |  |  | 174 | X_ViC_06 | Undergoing many transformations in social relationships. |
|  |  |  | 175 | X_ViC_07 | Undergoing many transformations in organizational and management methods. |
|  | **Cultural Preservation** | Environmental feature | 176 | M_ViC_043 | Houses of varying ages are present. |
|  |  | Social feature | 177 | X_ViC_012 | It is a long-established settlement. |
|  |  |  | 178 | X_ViC_024 | Geographically, the urban village lies within the city, yet people still maintain traditional activities in their daily lives. |
|  | **Migrant and Ethnic Integration** | Environmental feature | 179 | M_ViC_046 | The original traditional rural features of the village have changed significantly over time. |
|  |  | Social feature | 180 | X_ViC_013 | The resident population includes indigenous people. |
|  |  |  | 181 | X_ViC_014 | The resident population includes migrants from rural areas. |
|  |  |  | 182 | X_ViC_015 | The resident population includes migrants from urban areas. |
|  |  |  | 183 | X_ViC_04 | Traditional social norms are still maintained. |
|  | **Social and Spatial Cohesion** | Environmental feature | 184 | M_ViC_01 | It is a village enclosed by a city or urban area, yet retains distinct social characteristics and settlement forms typical of rural communities. |
|  |  |  | 185 | M_ViC_018 | Most urban villages still preserve core values that foster community bonds through religious structures and communal spaces such as village temples, pagodas, shrines, village ponds, markets, etc. |
|  |  |  | 186 | M_ViC_047 | The village’s original road network is largely preserved and has been upgraded. |
|  |  |  | 187 | M_ViC_053 | Public spaces in the village continue to host traditional activities on a regular basis. |
|  |  |  | 188 | M_ViC_060 | Renovated urban villages are former rural villages that have been converted into urban land through urban expansion. |
|  |  |  | 189 | M_ViC_070 | Public facilities such as community halls and churches, temples, and pagodas are places where village residents regularly engage in community activities. |
|  |  | Institutional and governance feature | 190 | TQ_ViC_033 | The urban village is a distinctive area within the city. |
|  |  | Social feature | 191 | X_ViC_025 | Positive social aspects present in the urban village are also essential in designing sustainable communities. |
|  | **Sustainable Economic Development** | Economic feature | 192 | K_ViC_010 | Over time, the density and land use value of urban villages increase, enhancing job opportunities for migrants |
|  |  |  | 193 | K_ViC_013 | Some urban villages have integrated creative elements and the creative industry into their development |
|  |  |  | 194 | K_ViC_017 | There are business initiatives based on tourism activities |
|  |  |  | 195 | K_ViC_021 | Residents have mixed incomes derived from both agricultural and non-agricultural economic activities |
|  |  |  | 196 | K_ViC_06 | Villagers and migrant workers have formed mutually beneficial economic partnerships |
|  |  | Institutional and governance feature | 197 | TQ_ViC_011 | Residents have the legal right to use their land. |
|  |  |  | 198 | TQ_ViC_025 | Closely related to institutional changes regarding collective and individual land ownership and usage rights. |
|  |  | Social feature | 199 | X_ViC_018 | It is a place that preserves cultural and humanitarian values and has significant urban economic potential. |
|  | **Urban and Agricultural Integration** | Economic feature | 200 | K_ViC_012 | Due to urbanization, urban villages face a reduction in agricultural land and activities and must adapt to new types of economic activities |
|  |  |  | 201 | K_ViC_09 | Urban villages have an agricultural past |
|  |  | Environmental feature | 202 | M_EX_2 | Currently, residents typically live in garden houses. |
|  |  |  | 203 | M_ViC_044 | Inherits the characteristic of having agricultural land within the city. |
|  |  |  | 204 | M_ViC_045 | Inherits the rural characteristic of garden houses. |
|  |  | Social feature | 205 | X_ViC_02 | There is a mix of rural and urban social characteristics. |
