# Supplementary material for: (Re)defining urban villages and their potential in sustaining local authenticity: A case study of Da Lat, Viet Nam
Source: PLoS One. 2026 Apr 3;21(4):e0345741. doi: 10.1371/journal.pone.0345741 (PMC13048443; doi:10.1371/journal.pone.0345741)
Supplement: S1 Text — (DOCX) [file pone.0345741.s012.docx]

| **S1 File. Code for criteria modeling using LDA**  Replace the link:  df=pd.read_csv('/content/ID_after_round_1_description_EN.csv')  by:  **S1 Dataset. OVs set after Round 1** |
| --- |
| **S2 File. Code for AHP and addressing inconsistencies using EMO**  Replace the link:  url = "https://docs.google.com/spreadsheets/d/1xi4Ff5_Wrn37I7VsXiYj7wP7aoPUVokPyBeBcLiXs6A/export?format=csv"  by:  **S3 Dataset. Expert AHP matrices** |
| **S3 File. Code for visualising number and distribution of Ovs**  Replace the link:  url = "https://docs.google.com/spreadsheets/d/14MrzpCvJFQynvnL50w-SIoCJWsi5i-idCUoxifGD2n4/export?format=csv"  by:  **S2 Dataset. Finalised OVs** |
| **S4 File. Code for villages assessments**  Replace the link:  url_OVs = "https://docs.google.com/spreadsheets/d/14MrzpCvJFQynvnL50w-SIoCJWsi5i-idCUoxifGD2n4/export?format=csv"  url_dalat = "https://docs.google.com/spreadsheets/d/1TnJgwqN5oV-d55RF13GNeH9m26gUZg5vbhoV-dNdtFY/export?format=csv"  url_vicinity = "https://docs.google.com/spreadsheets/d/1zLVOPzvEQ3YFgicTKHKHBoF2RRUMDT_36TuR7Kvhpvg/export?format=csv"  url_GUV = "https://docs.google.com/spreadsheets/d/1ZWilS5KMrHjgyvf7GiBV-nTebiEy-NFnBmGGHavZxTg/export?format=csv"  correspondingly by:  **S2 Dataset. Finalised Ovs**  **S4 Dataset. Scoring of villages in Da Lat**  **S5 Dataset. Scoring of villages in the vicinity**  **S6 Dataset. Scoring of the Green Urban Village** |
